# Intelligent nanovesicle for remodeling tumor microenvironment and circulating tumor chemoimmunotherapy amplification

**DOI:** 10.1186/s12951-024-02467-8

**Published:** 2024-05-16

**Authors:** Manxiu Huai, Yingjie Wang, Junhao Li, Jiaxing Pan, Fang Sun, Feiyu Zhang, Yi Zhang, Leiming Xu

**Affiliations:** 1 Department of Gastroenterology Xinhua Hospital, Shanghai Jiaotong University School of Medicine, Shanghai, 200092 China; 2 Nano Science and Technology Institute, University of Science and Technology of China, Suzhou, 215123 China; 3 Department of Nuclear Medicine, Shanghai Changhai Hospital, Shanghai, 200433 China

**Keywords:** Pancreatic ductal adenocarcinoma, Chemoimmunotherapy amplification, 5-FU, Nanovesicle, Kyn-AHR axis

## Abstract

Imperceptible examination and unideal treatment effect are still intractable difficulties for the clinical treatment of pancreatic ductal adenocarcinoma (PDAC). At present, despite 5-fluorouracil (5-FU), as a clinical first-line FOLFIRINOX chemo-drug, has achieved significant therapeutic effects. Nevertheless, these unavoidable factors such as low solubility, lack of biological specificity and easy to induce immunosuppressive surroundings formation, severely limit their treatment in PDAC. As an important source of energy for many tumor cells, tryptophan (Trp), is easily degraded to kynurenine (Kyn) by indolamine 2,3- dioxygenase 1 (IDO1), which activates the axis of Kyn-AHR to form special suppressive immune microenvironment that promotes tumor growth and metastasis. However, our research findings that 5-FU can induce effectively immunogenic cell death (ICD) to further treat tumor by activating immune systems, while the secretion of interferon-γ (IFN-γ) re-induce the Kyn-AHR axis activation, leading to poor treatment efficiency. Therefore, a metal matrix protease-2 (MMP-2) and endogenous GSH dual-responsive liposomal-based nanovesicle, co-loading with 5-FU (anti-cancer drug) and NLG919 (IDO1 inhibitor), was constructed (named as ENP919@5-FU). The multifunctional ENP919@5-FU can effectively reshape the tumor immunosuppression microenvironment to enhance the effect of chemoimmunotherapy, thereby effectively inhibiting cancer growth. Mechanistically, PDAC with high expression of MMP-2 will propel the as-prepared nanovesicle to dwell in tumor region *via* shedding PEG on the nanovesicle surface, effectively enhancing tumor uptake. Subsequently, the S-S bond containing nanovesicle was cut *via* high endogenous GSH, leading to the continued release of 5-FU and NLG919, thereby enabling circulating chemoimmunotherapy to effectively cause tumor ablation. Moreover, the combination of ENP919@5-FU and PD-L1 antibody (αPD-L1) showed a synergistic anti-tumor effect on the PDAC model with abdominal cavity metastasis. Collectively, ENP919@5-FU nanovesicle, as a PDAC treatment strategy, showed excellent antitumor efficacy by remodeling tumor microenvironment to circulate tumor chemoimmunotherapy amplification, which has promising potential in a precision medicine approach.

**Supplementary Information:**

The online version contains supplementary material available at 10.1186/s12951-024-02467-8.

## Introduction

Pancreatic ductal adenocarcinoma (PDAC) is a kind of malignant tumors whose 5-year survival rate is less than 5%, and has fourth highest the mortality rate among all malignant tumors [1, 2], mainly due to its high concealment, complex heterogeneity and serious off-target effect on frontline clinical drugs, etc. In addition, studies found that PDAC, as a hypoglycemic tumor with a dense extracellular matrix, will severely impede the infiltration of immune cells and discourage drug distribution and entry [3]. Therefore, PDAC with special physiological characteristics will bring great obstacles to the development of treatment, and it is difficult to achieve satisfactory clinical outcomes in original treatment strategies. Implausibly, current PDAC chemotherapy, such as gisitabine, is only available for a one-year survival rate of no more than 20% as a first-line treatment [4]. It is therefore imperative to develop an effective PDAC treatment strategy and explore potential cellular mechanisms, as well as how to effectively improve drug distribution and bioavailability, and break down the suppressive immune microenvironment play a significant role in drug-mediated treatment of PDAC.

Normally, in addition to cancer cells, complicated and plentiful matrix, innate and adaptive immune cells, etc. jointly establish complex tumor ecological microenvironment [5–7]. This complex cell group forms a cooperative and competitive cycle that supports tumor proliferation, progression, metastasis, and immune evasion, leading to tumor deterioration. Therefore, ameliorating tumor immunosuppressive microenvironment (TIM) is an important link in the optimizing anti-tumor therapy. Extensive research has confirmed that amino acids are an important source of energy for tumor cells, causing tumor tissues to exhibit metabolic remodeling of a range of metabolites, such as kynurenine (Kyn) and ROS [7, 8]. Meanwhile, these toxic metabolites are often involved in the formation of inhibitory immunity. Tryptophan (Trp) and related metabolic derivatives, as cellular energy supply substances, are involved in many processes of physiology and signaling molecules to transmit biological information. Many kinds of enzymes, like, indometamine-2,3-dioxygenase 1 (IDO1) [9], lysine acetyltransferase (KATs) [10], and interleukin-4 inducing gene 1 (IL4I1) [11], can react with Trp molecule to generate various derivatives (such as Kyn, IP3, etc.), and exert biological effects, involving inflammation, immune response, and excitatory neurotransmission, etc [12]. . Regrettably, it is important to point out that Kyn is an essential ligand for aryl hydrocarbon receptors (AHR), which will lead to activity of the Kyn-AHR axis, thereby up-regulating the gene expression of the unfavorable factors on the tumor therapy, such as TCDD-inducible poly-ADP-ribose polymerase (Tiparp) and cytochrome P450 1B1 (CYP1b1), which further expand metastasis, invasion, and immunosuppression of tumor [13, 14]. Given that Kyn-AHR axis has been shown to be associated with of many kinds of tumor immunosuppression [14–16], how to overcome or improve relational therapeutic deficiencies is a challenge for the tumor therapy.

Currently, FOLFIRINOX chemotherapy, like 5-fluorouracil (5-FU), oxaliplatin, irinotecan, and leucovorin, as the first-line treatment drugs, has acquired impressive treatment results [17, 18]. Therein, 5-FU, is a chemotherapeutic drug that inhibits DNA synthesis to produce tumor suppression, which is widely used in the treatment of multiple solid tumors, especially in PDAC treatment [19, 20]. Moreover, 5-FU can excite the autoimmune system through the 5-FU induced-immunogenic cell death (ICD) effect, such as calreticulin (CRT) eversion, high mobility group protein 1 (HMGB1) efflux, etc., activating dendritic cells (DCs) and increase T cells infiltration, as well as activation tumor local immunity [21]. During this process, interferon-γ (IFN-γ) from activated T cells will show an increasing trend and play a tumor immunodepression effect *via* the two ways, i.e., the regulation of the classic first type of major histocompatibility complex (MHC-I) to inhibit natural killer (NK) cells and the inhibition of CD8 + T cells by upregulating non-classical MHC-I molecules Qa-1b [22]. In addition, the secretory IFN-γ also up-regulates the expression of IDO1 and leads to the activation of the Kyn-AHR axis, thereby creating a suppressive immune microenvironment [23, 24]. Therefore, optimizing the regulation of metabolic pathways to overcome tumor resistance to drugs and improve tumor immune microenvironment is supposed to being a potential alternative to curb tumor escape. Given the high expression of IDO1 in PDAC [25] and the low therapeutic efficacy of first-line therapies, thus, how to use metabolic ways to achieve the amplified therapeutic effect by chemo-immunotherapy has been explored.

**Scheme 1:**
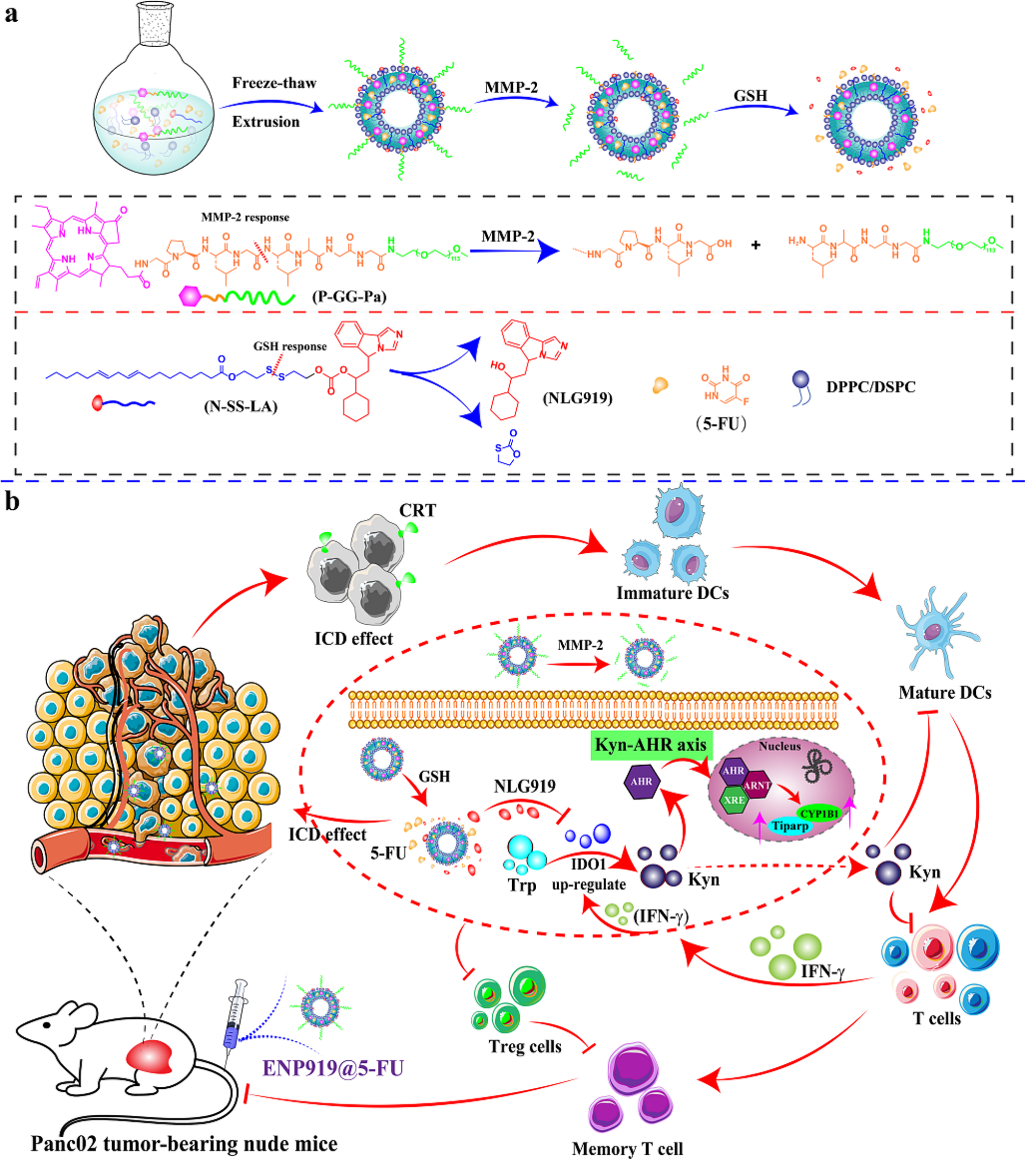
(**a**) Schematic illustration of the MMP-2-sheddable and GSH-responsive nanovesicle for circulating tumor chemoimmunotherapy amplification. (**b**) The mechanism of ENP919@5-FU mediated chemo-immunotherapy to treat PDAC by remodeling tumor microenvironment

Currently, liposome-based nano-delivery systems (L-NDSs) are widely used due to their significant biocompatibility, implified preparation, and easy drug portability, especially in the field of cancer treatment [26]. In addition, specific physiological microenvironments based on tumor tissue, such as high doses of metal matrix protease-2 (MMP-2), abundant bio-endogenous glutathione (GSH) in tumors, etc [27]., provide trigger points for responsive L-NDSs in the tumor site, compared to normal tissue. MMP-2 is a kind of secreted protein that exists in the extracellular matrix and is involved in tumor invasion, and is therefore a target of anti-tumor drugs [28, 29]. Herein, a MMP-2/GSH dual-responsive liposome-based nanovesicle (ENP919@5-FU) was rationally fabricated, where 5-FU (chemotherapy drugs) and NLG919 (IDO1 inhibitors) co-loaded in nanovesicle, by reversing chemotherapy-mediated inhibition of the immune environments to achieve ICD amplification, further thereby circulating amplification chemo-immunotherapy. The fabrication and the principle of circulating chemo-immunotherapy amplification by remodeling tumor microenvironment was illustrated in Scheme 1. In the preparation process, the introduced as-prepared mPEG-decorated PPa component into nanovesicle was proposed, on the one hand, the utilization of real-time fluorescence imaging, on the other hand, as a lipidic cornerstone to stabilize the stability of lipid-based nanovesicle. Meanwhile, the containing S-S linoleic acid-embellished NLG919 was first manufactured and successfully inserted into lipid-based nanovesicle. The ENP919@5-FU nanovesicle was easily captured by high expression MMP-2 enzymes in the tumor site, resulting in relative size changes-dependent accumulation and retention in tumor tissue, thereby effectively enhancing tumor cellular uptake. Meanwhile, in high standard of endogenous GSH, NLG919 and 5-FU were released from nanovesicle and exposed 5-FU would be effective in inducing cell death and ICD effects. The 5-FU-mediated ICD effect activated the biological immune systems in tumor tissues, however, IFN-γ from T cells could re-persuade the Kyn-AHR axis activation, leading to poor efficiency of tumor treatment. Fortunately, the release of NLG919 was responsible for inhibiting the Kyn-AHR channel, and restated the reversal of the tumor immunosuppression microenvironment, thus realizing intelligent nanovesicle-mediated chemoimmunotherapy cycle amplification in PDAC. Additionally, ENP919@5-FU nanovesicle combined with PD-L1 antibodies could effectively and reasonably prevented tumor recurrence, through activating DCs, and initiating cytotoxic T lymphocytes, as well as forming the long-term immune memory effect immunity, which provides a simple and promising approach to treat metastatic cancers and prevent tumors.

## Results and discussion

### NLG919 improves 5-FU-induced TIM

**Fig. 1:**
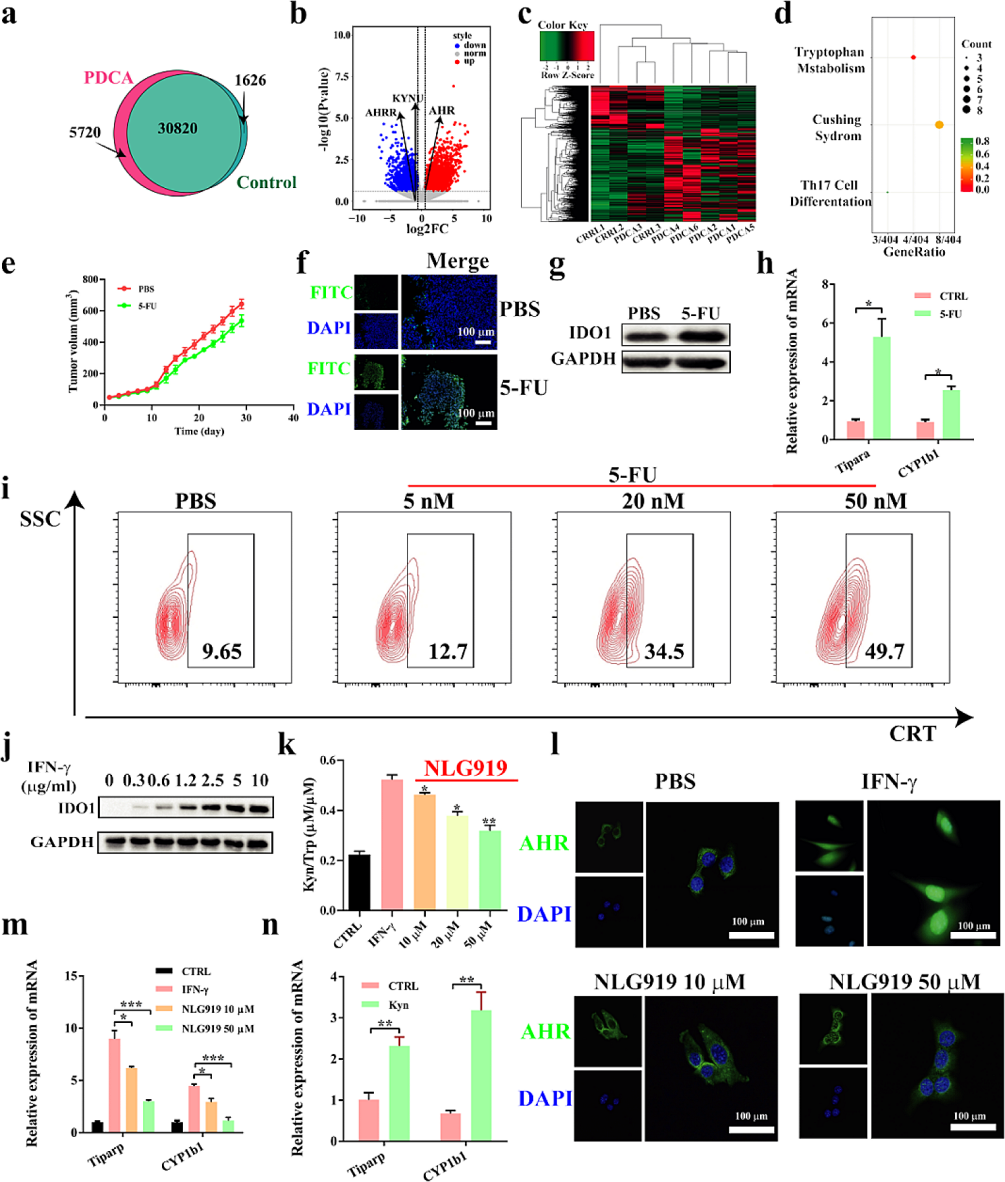
NLG919 improve 5-FU-induced tumor-suppressive immune microenvironment. (**a**) Venn diagram showed the differential gene expression between pancreatic cancer tissue and paracancerous tissue samples. (**b**) Volcanic maps showed the difference of gene expression levels in pancreatic cancer tissue and paracancer tissue. (**c**) Cluster analysis was performed on the sequencing results. (d) Bubble map indicated that the Kyn-AHR pathway was activated in pancreatic cancer. (**e**) Tumor growth curves. (**f**) TUNEL staining of each group after the treatments. (**g**) 5-FU-induced up-regulation of IDO1 expression in Panc02-bearing C57 mice. (**h**) qPCR detected the expression of Tiparp and CYP1b1 in Panc02-bearing C57 mice. (**i**) IF detected CRT eversion in Panc02 cells. (**j**) Western blot detected the expression of IDO1 expression in Panc02 cells. (**k**) ELISA detected the Kyn/Trp ratio mediated by IFN-γ in Panc02 cell. (**l**) FCM detected the location of AHR in Panc02 cell. (**m**) qPCR detected the expression of Tiparp and CYP1b1 Panc02 cells. (**n**) qPCR detected the expression of Tiparp and CYP1b1 in BMDCs. Statistical analysis was performed by unpaired *t*-test, *, *p* < 0.05; **, *p* < 0.01; ***, *p* < 0.001; ****, *p* < 0.0001

Firstly, the RNA sequencing was performed on human pancreatic cancer tissue (PDAC) and paracancerous tissue samples from general surgery department of Xinhua hospital (approval number: XHEC-C-2021-096-1). The analysis showed that there were specific differences between PDAC and adjacent tissue in partial gene expression. As shown in Fig. 1a, a total of 30,820 genes was co-expressed in pancreatic and paracancer tissue, and 5720 and 1626 genes were expressed only in pancreatic and paracancer tissue, respectively. In addition, we also conducted cluster analysis of gene expression in PDAC and paracancerous tissue to further analyze the differences in expression (Fig. 1b and c). Moreover, tryptophan metabolism-related pathways in PDAC were significantly up-regulated compared to paracancer tissue, while tryptophan antagonistic pathways, such as aryl hydrocarbon receptor repressor (AHRR) and kynureninase (KYNU) [30], were displayed a downward trend (Fig. 1d). Therefore, these data indicated that tryptophan and its metabolites have some effect on the development and outcome of pancreatic cancer, and intervention in tryptophan metabolism may change the prognosis of PDAC.

As mentioned before, 5-FU, as a frequently-used FOLFIRINOX chemotherapy drug, is also often chosen for PDAC in clinic. However, 5-FU provides a great narrow therapeutic effect and a poor prognosis [17]. Further exploration of 5-FU-mediated PDAC treatment is therefore of great significance, and a great platform for 5-FU-based combination-therapy. Simply, free 5-FU drug (50 mg/kg) was injected into Panc02 tumor-bearing C57 mice three times (at 1st, 4th and 7th day, respectively) *via* the tail vein. The single-agent 5-FU-treated mice with Panc02 tumor showed significant tumor inhibition effect, compared to PBS group (Fig. 1e). At the same time, 5-FU induced apoptosis was significantly observed through immunofluorescence images (Fig. 1f**)**, indicating that free 5-FU could inhibit tumor growth. Additionally, 5-FU could induced CRT eversion in Panc02 cells with a concentration-dependent increase (Fig. 1i), indicating its potential to induce ICD effects [31]. Inspired by the tryptophan metabolism-related tunnel assumption, we conducted a series of experiments to assess the relationship between tumor recurrence and the activation of Kyn-AHR axis, as well as the role of IFN-γ in this process. Collected results found that an increasing IFN-γ expression (Fig. S2a) in the 5-FU-treated Panc02 cells, meanwhile, the expression level of IDO1 performed an upward trend (Fig. 1g). Additionally, the ratio of Kyn/Trp (Fig. S2b, 2c, 2d) and the expression of Tiparp and CYP1b1 (Fig. 1h) also increased. These data indicated that tumor recurrence may be associated with the activation of the Kyn-AHR axis caused by 5-FU application, while IFN-γ acts as a bridge role in this procedure [13, 32, 33]. Therefore, we hypothesized that inhibiting the activation of Kyn-AHR axis might prevent tumor recurrence secondary to 5-FU administration, and restrain the formation of TIM, hindering tumor metastasis capacity.

**Fig. 2:**
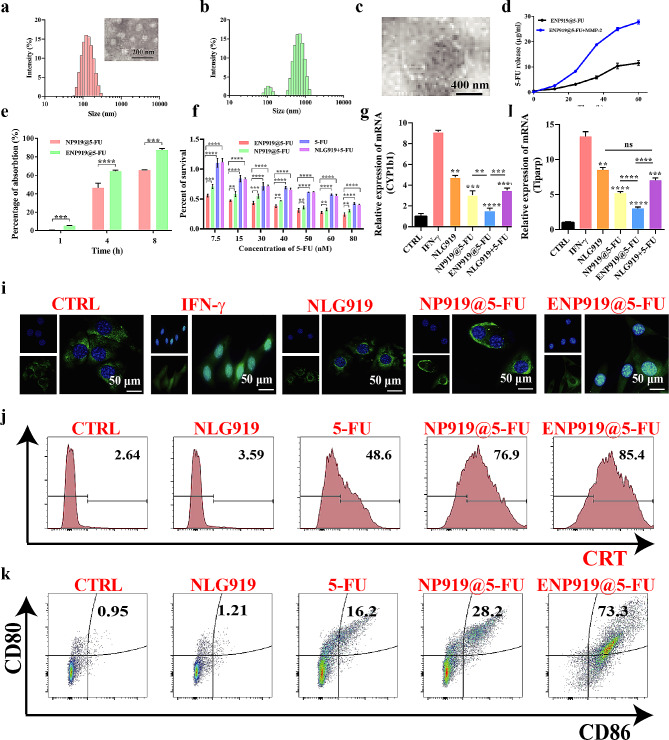
Characterization and biological function of ENP919@5-FU (**a**) particle size distributions and TEM image of ENP919@5-FU. (**b**, **c**) Dissociation behaviour of ENP919@5-FU under MMP-2 enzyme co-incubation for 48 h by DLS detection (**b**) and TEM technology (**c**). (**d**) The release of 5-FU drug from ENP919@5-FU by HPLC detecting. (**e**) FCM detected the uptake of ENP919@5-FU (130.8 µg/mL) and NP919@5-FU (130.8 µg/mL) in vitro. (**f**) CCK-8 detected the cytotoxicity of ENP919@5-FU, NP919@5-FU, 5-FU and NLG919 + 5-FU in vitro. (**g**, **h**) qPCR detected the expression of CYP1b1 and Tiparp in vitro. (**i**) IF detected the location of AHR in vitro. (**j**) FCM detected CRT in vitro. (**k**) FCM detected the expression of CD11c, CD80, and CD86 on BMDCs. Statistical analysis was performed by unpaired *t*-test, *, *p* < 0.05; **, *p* < 0.01; ***, *p* < 0.001; ****, *p* < 0.0001

NLG919 is an inhibitor of the IDO pathway, which can effectively alleviate TIM and enhance the curative effect of tumor. As shown in Fig. 1j and S3, the expression of IDO1 in Panc02 cells showed a concentration-dependent increase with IFN-γ introduction, suggesting IFN-γ could stimulate the production of IDO1. Furthermore, NLG919 significantly could reduce the Kyn/Trp ratio (Fig. 1k), and further inhibited the nuclear translocation of AHR (Fig. 1l, S3), leading to the expression level of Tiparp and CYP1b1 showed a downward trend (Fig. 1m). Subsequently, the immunosuppression effect of Kyn signal was measured by the use of mouse bone-marrow-derived dendritic cell (BMDC) technology. Kyn administration up-regulated the expression of Tiparp and CYP1b1 in mouse BMDC significantly, indicating that the activation of the Kyn-AHR axis could create TIM (Fig. 1n). Overall, these data confirmed that the NLG919 can improve potently the 5-FU-induced TIM by inhibiting Kyn-AHR axis, thereby inhibiting tumor growth and neoplasm metastasis, which will provide a fresh thinking for chemoimmunotherapy amplification to treat tumors.

### Characterization and biological functions of ENP919@5-FU nanovesicle

Inspired by the specificity of the tumor’s physiological microenvironment, such as higher-dose GSH and MMP-2 enzyme [27–29] the design of tumor-responsive nanovesicle to increase drug delivery capacity and continuous release at the location of tumor provides a steadfast guarantee. Prior to this, we successfully synthesized the MMP-2 enzyme-responsive PEG-modified PPa fluorescent probe (P-GG-Pa) and the containing S-S linoleic acid grafting IDO inhibitor (N-SS-LA) by ^1^H NMR spectroscopy detection (Fig. S4, S5, S6, S7, S8, S9, S10). To verify the dissociation of GSH-mediated N-SS-LA, the GSH response-relevant experiments were carried out and tested by HPLC technology. As shown in Fig. S14, the N-SS-LA molecule showed a time-dependent degradation ability, with almost 80% degradation in about 6 h, inducting the successful synthesis of the GSH-responsive N-SS-LA molecule. Subsequently, the as-prepared blank ENP919 nanovesicle, consisting of DPPC, DSPC, N-SS-LA and P-GG-PA with a volume ratio of 3:1:1:3 (v/v), was prepared by traditional membrane method [34]. Other distinct nanovesicles, like, NP919@5-FU and ENP919@5-FU, were also constructed according to similar approaches (Scheme 1). The hydrodynamic diameters and PDI values of ENP919@5-FU nanovesicle, NP919@5-FU nanovesicle, and ENP919 nanovesicle were 133.02 ± 0.047 nm and 0.069 ± 0.022, 136.47 ± 2.37 nm and 0.073 ± 0.032, 131.32 ± 0.622 and 0.047 ± 0.012, respectively, *via* the dynamic light scattering (DLS) test, meanwhile, the spherical morphology of the series prepared nanovesicles were displayed by TEM image (Fig. 2a, S11, S12). Additionally, it was obvious that the blank ENP919 nanovesicle was smaller than the 5-FU-loaded nanovesicle owing to 5-FU was successfully encased in nanovesicle. Moreover, these nanovesicles demonstrated significant stability in PBS, maintaining low range fluctuations on hydrodynamic diameters and PDI values for 3 days, mainly due to the surface PEG chain plays an important role in forming hydrophilic corona (Fig. S13). Interestingly, ENP919@5-FU was cracked and unstable when the presence of MMP-2 enzyme, through DLS and TEM detection (Fig. 2b and 2c). This phenomenon was mainly due to surface PEG-decorated ENP919@5-FU was cut off by MMP-2 enzyme, resulting in its loss of protection of hydrophilic PEG layer, which provides an opportunity for the enrichment of ENP919@5-FU at the tumor site with the over-expressed MPP-2 enzyme. Besides, the encapsulation efficiency and drug loading rate of 5-FU in ENP919@5-FU and NP919@5-FU were measured as 40.2%, 34.9% and 38.5%, 41.3%, respectively, *via* HPLC detection. Meanwhile, NLG919, connected with linoleic acid as an important component of nanovesicle, whose encapsulation efficiency and the loading rate was 100% and 10.3%, respectively. After that, 5-FU release curve from ENP919 nanovesicle was evaluated, and 5-FU showed a trend of continuous release in introduction of MMP-2 enzyme, and even after 24 h, the cumulative release of 5-FU reached 65% (Fig. 2d).

By querying the database Gepia (http://gepia.cancer-pku.cn/) and looking for previously reported literature [28, 29], we determined that MMP-2 is highly expressed in pancreatic cancer (**Fig. ****S1****a**). Subsequently, we commissioned the ABIOCENTER company to customize the MMP-2-KD Panc02 cells to investigate ENP919@5-FU nanovesicle phagocytosis. As shown in **Fig. ****S1****b and 1c**, after the uptake rate of liposomes was significantly reduced in MMP-/- Panc02 cells, compared to WT group. Thus, it could be inferred that nanovesicle with MMP-2 responses is more conducive to tumor uptake. Thus, the efficiency of cell phagocytosis is an important factor in L-NDSs, which is recognized as a cornerstone to improve L-NDSs-mediated treatment [33]. Therefore, cell uptake behavior of ENP919@5-FU was investigated in Panc02 cells in vitro. The findings showed cell uptake efficiency of ENP919@5-FU was higher than NP919@5-FU, mainly due to the removal of surface PEG fragments in the presence of MMP-2 enzyme (Fig. 2e).

The cytotoxicity of the ENP919@5-FU was evaluated on Panc02 cells through CCK-8 assay. The ENP919-treated Panc02 cells displayed a negligible cytotoxicity fluctuation as the concentration increase, even reaching 50 µg/mL, demonstrating significant biocompatibility (**Fig. S15a**). The free NLG919 showed low cytotoxicity to Panc02 cells through the CCK-8 assay (**Fig. S15b**). Subsequently, the free NLG919 was co-incubated with 5-FU and the cytotoxicity did not fluctuate compared to free 5-FU treated group, indicating that the NLG919 had little toxicity. Meanwhile, the free NLG919 did not displayed 5-FU synergistic enhancement of the effects of tumor therapy. A trend of concentration-dependent cytotoxicity was displayed on 5-FU loaded nanovesicle (ENP919@5-FU and NP919@5-FU), owing to the release of 5-FU from nanovesicle in vitro. Nevertheless, the cell viability of ENP919@5-FU nanovesicle was lower than that of NP919@5-FU, mainly due to MMP-2 enzyme acceleration 5-FU release from ENP919@5-FU nanovesicle, consistent with the release profile of 5-FU. Encouragingly, the cytotoxicity of nanovesicle-carrying 5-FU was much higher than free 5-FU drug. Theoretically, on the one hand, released NLG919 assists 5-FU to amplify therapeutic effects, on the other hand, it may be the devouring of nanovesicle-enhanced the anti-tumor effect of 5-FU (Fig. 2f).

Next, we investigated the mechanism by which ENP919@5-FU reverses 5-FU-induced TIM. Free IFN-γ effectively activated the Kyn-AHR axis, as well as the up-regulation of CYP1b1 and Tiparp expression. However, compared to NP919@5-FU, due to the differences in cell uptake efficiency, the activation of Kyn-AHR axis was more effectively reversed by ENP919@5-FU, which specifically manifested as AHR nuclear translocation reduction and the suppression of CYP1b1 and Tiparp expression (Fig. 2g, 2h and 2i). These data indicated that although 5-FU administration could active the Kyn-AHR axis, leading to the failure of tumor therapy, the MPP-2/GSH response-based ENP919@5-FU nanovesicle could be effectively reversed.

5-FU inducing the production of ICD in tumor cells has been explored and therefore can be effective in stimulating an adaptive anti-tumor immunity, where CRT eversion serves as a key ICD indicator. By FCM detection, ENP919@5-FU nanovesicle (85.4%) could induce a stronger ICD effect on Panc02 cells, compared to free 5-FU (48.6%) or NP919@5-FU nanovesicle (76.9%), owing to MMP-2 enzyme-mediated ENP919@5-FU nanovesicle instability, which increases cell uptake capability and drug release behavior. In contrast, the ICD induction almost no appeared through NLG919-treated Panc02 cells (3.59%), compared to the control group (2.64%) (Fig. 2j). The increased percentage of CRT eversion provided the possibility for ENP919@5-FU nanovesicle-activated immunotherapy.

Generally, the enhanced immunogenicity of Panc02 cells will promote the maturation of bone-marrow-derived dendritic cells (BMDCs). Therefore, to build the maturation DC cells, the routine process was 24 h of treatment of Panc02 cells with different samples (such as PBS, ELN919, 5-FU, NP919@5-FU and ENP919@5-FU), followed by another 24 h of incubation with BMDCs from C57 mice. The relevant indicators (CD11c, CD80 and CD86) maturation [27, 35] of DCs were collected and detected by FCM. A weak DC maturation induction effect in ENP919 treatment (1.21%) was displayed mainly due to ENP919 could not effectively kill Panc02 cells to induce ICD effect. However, containing 5-FU could drive Panc02 cells to expose its antigens, which induced DC maturation with a limit outcome (16.2%). Surprisingly, ENP919@5-FU with MMP-2/GSH dual-responses showed a more efficient DC maturation induction (73.3%), compared to NP919@5-FU treatment (28.2%) (Fig. 2k), indicating that the significant excitation immunity systems.

### Tumor suppression effect of ENP919@5-FU in vivo

**Fig. 3:**
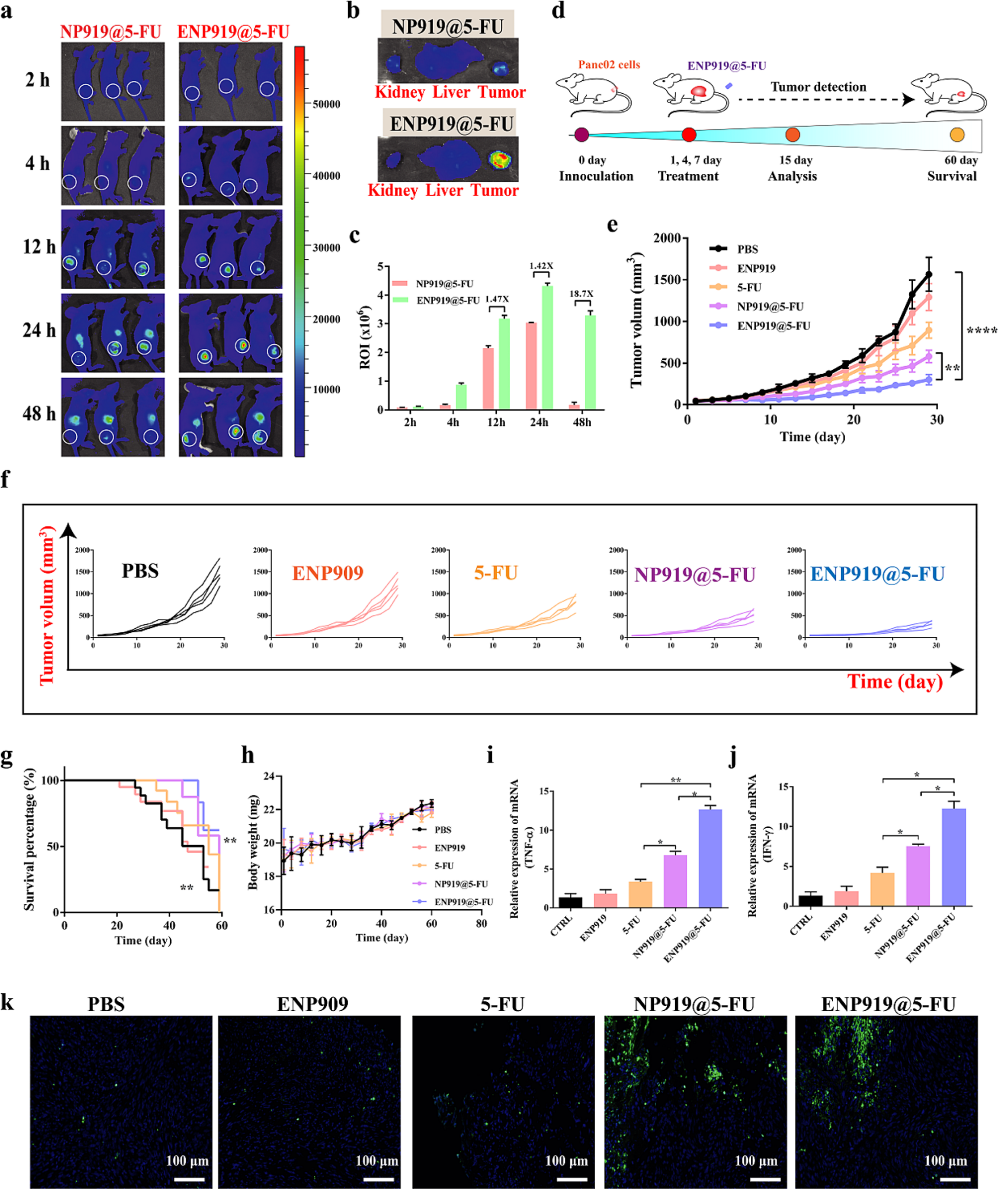
The tumor suppression effect of ENP919@5-FU in vivo. (**a**) IVIS small animal imaging system-based analysis of the distribution of ENP919@5-FU and NP919@5-FU in nude mice at different time points. (**b**) Fluorescent images of vital organs (kidney and liver) and tumor in mice *ex vitro*. (**c**) Analysis of tumor ROI intensity at each time point. (**d**) Schematic of mice treatment. (**e**, **f**) Tumor growth curve of mice in each group. (**g**) Survival curve of mice in each group. (**h**) Changes in weight in mice. (**i**, **j**) qPCR analysis of TNF-α expression (**i**) and IFN-γ expression (**j**) in Panc02-bearing C57 mice. (**k**) Tunel staining of mouse tumors. Statistical analysis was performed by unpaired *t*-test and one-way ANOVA, *, *p* < 0.05; **, *p* < 0.01; ***, *p* < 0.001; ****, *p* < 0.0001

To comprehend the distribution of the ENP919@5-FU nanovesicle in vivo, the pre-established Panc02 tumor-bearing nude mice were randomly divided into two groups, such as NP919@5-FU (containing PPa 5 mg/kg) group and ENP919@5-FU (containing PPa 5 mg/kg) group, and used the IVIS small animal imaging system to real time tracking *via* tail vein injection. Fluorescence imaging of NP919@5-FU administration mice demonstrated an unsatisfactory accumulation at the tumor site and gradually declined due to its rapid blood clearance from the tumor tissue (Fig. 3a). In contrast, at 12 h post-injection, the ENP919@5-FU accumulation in the tumor sites was detected with a 1.47-fold higher than that in NP919@5-FU group (Fig. 3c). Thus, ENP919@5-FU gradually accumulated at the tumor site and reached the highest intensity at about 24 h after injection. Meanwhile, after 48 h, important organs (kidney and liver) and tumors were removed from treated-mice for organ fluorescence imaging ex vivo and tissue fluorescence imaging (Fig. 3b). In addition, results found that the ENP919@5-FU-treated group had a stronger PPa fluorescence, compared to the NP919@5-FU group, due to MPP-2-mediated cleavage of the PEG corona (**Fig. S17**). As a result, ENP919@5-FU has specific tumor enrichment ability to enhance efficiency of treatment tumor.

Subsequently, the anti-tumor effect of ENP919@5-FU was assessed on the Panc02-bearing C57 mouse model. The entire process of experiment in Panc02-bearing C57 mice was shown in Fig. 3d. An evident increasing trend of mouse weight in each group was observed in treatment processes, indicating the living situation of mouse was not disturbed by samples administration (Fig. 3h). As expected, based on the tracking data of variation of tumor volume, we found that ENP919@5-FU could effectively impede tumor growth, compared to the PBS group. Other treatment groups, such as ENP919, 5-FU and NP919@5-FU, also showed a growth inhibition in Panc02 tumors, but limited therapeutic efficiency was collected compared to ENP919@5-FU administration, also further illustrating the superiority of ENP919@5-FU in mediate tumor therapy (Fig. 3e and 3f). As shown in Fig. 3g, ENP919@5-FU-administrated mice with Panc02 tumor showed a survival advantage during the 60-day period, indicating that the remarkable improvement of prognosis of mice. Unhappily, the remaining groups of mice did not show significant therapeutic benefits, such as ENP919, 5-FU and NP919@5-FU. Meanwhile, hematoxylin-eosin (H&E) staining (Fig. S19) and TUNEL staining (Fig. 3k, S16) also further displayed ENP919@5-FU significantly promoted apoptosis and necrosis in the tumor cells [36].

To investigate the ICD effect of ENP919@5-FU in vivo, the tumor tissues were detected by immunofluorescence images. The CRT eversion-induced strong green fluorescence intensity was shown through ENP919@5-FU treating, indicating that ENP919@5-FU could effectively induce ICD effect, activating the body immunity (Fig. S18). Meanwhile, by qPCR analysis, ENP919@5-FU also showed a highest expression level of TNF-α (Fig. 3i) and IFN-γ (Fig. 3j) in tumor tissues, compared with other treatment groups, demonstrating that recombinant nanovesicle combined with 5-FU drug could deadly activate the tumor immunity and improve the effect of tumor therapy.

Taken together, these data suggested that ENP919@5-FU, due to MMP-2-mediated the dissection of PEG part, as well as combination with 5-FU drug and NLG919 inhibitor to optimizing drug treatment effects, thus strengthening anti-tumor effects, was a novel treatment platform.

### Reconstruct tumor immune microenvironment

**Fig. 4:**
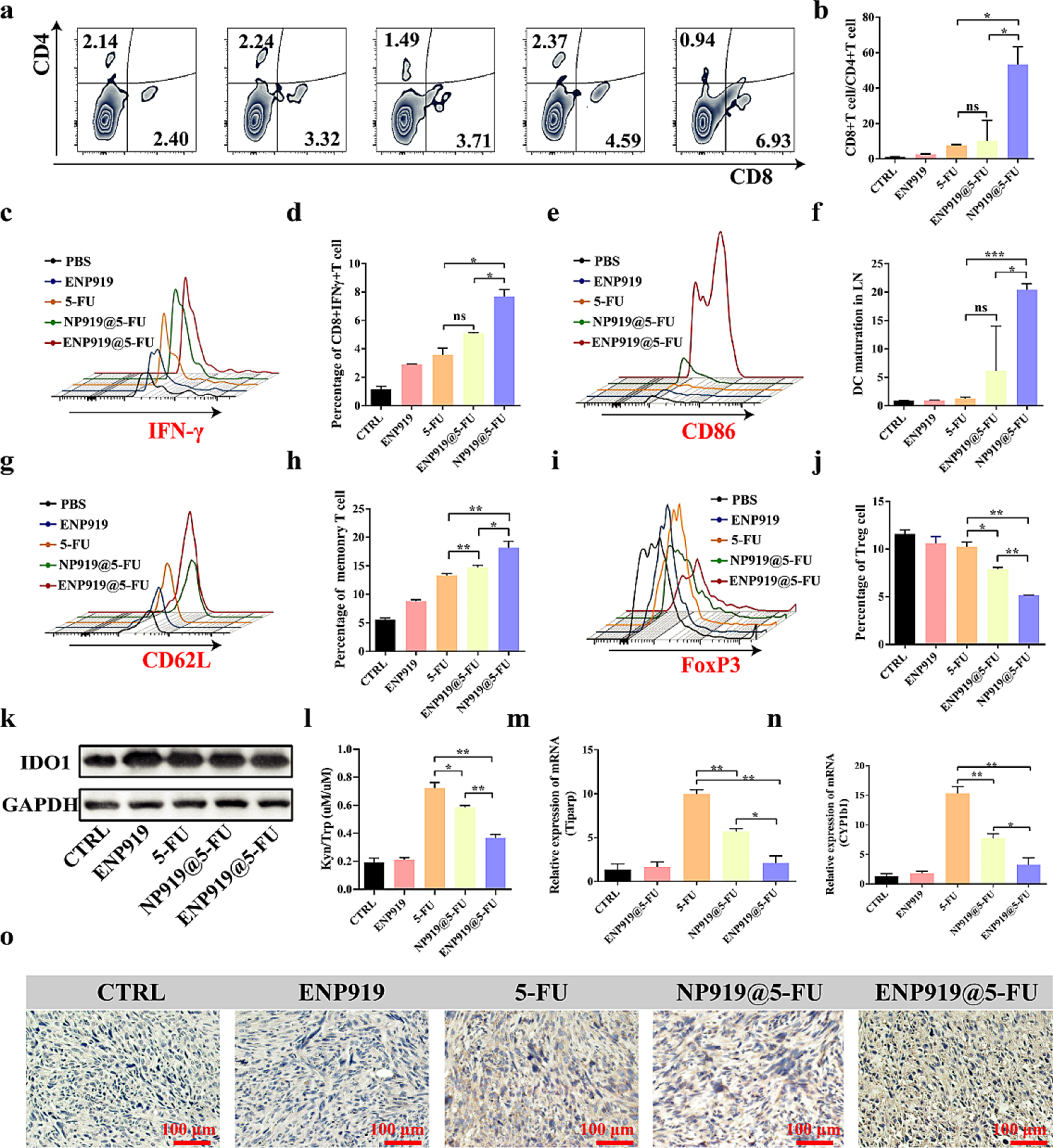
ENP919@5-FU improved tumor immune microenvironment. (**a**-**j**) Proportion and analysis of CD4/CD8 T cells, CD8 + IFN-γ + T cells, DCs, Treg cells, central memory T cells in tumor microenvironment or tumor-draining lymph nodes. (**k**) Western blot analysis of IDO1 expression in mouse tumors from each group. **l**) ELISA detection of the ratio of Kyn/Trp in Panc02-bearing C57 mice. (**m**-**n**) qPCR detected the gene expression of Tiparp and CYP1b1 in Panc02-bearing C57 mice. (**o**) IHC detection of AHR expression in Panc02-bearing C57 mice. Statistical analysis was performed by unpaired *t*-test, *, *p* < 0.05; **, *p* < 0.01; ***, *p* < 0.001; ****, *p* < 0.0001

According to the decreased recurrence rate of the primary tumors, we explored the mechanism of tumor suppression effect mediated by ENP919@5-FU. After injection for 15th day, subcutaneous tumors and tumor-draining lymph nodes were harvested and digested into a single-cell suspension to extract immune cells for FCM analysis. The low ratio of CD8 + T cells/CD4 + T cells for PBS group indicated that the tumors were a microenvironment that inhibited immunity [37]. In addition, the immunosuppressive environments were somewhat improved through NLG919, 5-FU and NP919@5-FU treatment, but with limited effects and even NLP919 did not work. Nevertheless, ENP919@5-FU intensely promoted the differentiation of CD8 + T cells in the tumor microenvironment, increasing the ratio of CD8 + T cells / CD4 + T cells (Fig. 4a and b). Moreover, a similar trend also obtained on the percentage of IFN-γ + CD8 + T cells in tumors by different samples managing, i.e., ENP919@5-FU induced optimal immune effect. However, NP919@5-FU did not show an advantage in increasing the propotion of IFN-γ + CD8 + T cells (Fig. 4c and d). Meanwhile, DC maturation, central memory T cell (TCM) differentiation and Treg cell level in tumor-draining lymph nodes of related treatments were detected. ENP919@5-FU-treated mice still played an outstanding effect on amelioration of DC (Fig. 4e and f) and TCM differentiation (Fig. 4g and h), which proving long-lasting anti-tumor immunity. Moreover, ENP919@5-FU significantly inhibited Treg cells differentiation and effectively broke down tumor immune suppression (Fig. 4i and j). Other-treated groups, including NLG919, 5-FU and NP919@5-FU treatment, showed weak capacity of immune activation, resulting in poor therapeutic outcomes, which coincided with the above obtained therapeutic results. To this end, we explored further the associated therapeutic mechanisms of immunosuppressive behaviors. As we already know 5-FU-induced tumor immunogenicity can be enhanced to invoke immune-systems and enhance DC maturity, but collected results are poor the performance of immune. Thus, we justifiably speculate that the pathways to immune activation are blocked based on the impact of cell-level assessment. Subsequently, although nanovesicles containing NLG919 did not prominently prevent the expression of IDO1 in tumors due to the stimulation of IFN-γ (Fig. 4k), both ENP919@5-FU and NP919@5-FU remarkably decreased Kyn/Trp ratio, compared to free 5-FU. (Figure 4l, m and n, S20). After that, the AHR expression in Panc02-bearing mice was investigated using IHC detection. As expected, it is ENP919@5-FU that led to nuclear translocation AHR noticeably suppressed due to a lower level of Kyn (Fig. 4o). Meanwhile, the Tiparp and CYP1b1 expression level in the administration of ENP919@5-FU restored to normal standards, suggesting that the poor prognosis of tumor was reversed, i.e., amplifying therapeutic effects.

Collectively, ENP919@5-FU reshaped immunosuppressive environment by Kyn-AHR axis suppression, contributing to enhanced T cell recruitment and activation and the formation of immune memory to improve anti-tumor effects.

### Combined αPD-L1 significantly inhibits tumor recrudescence and metastasis

**Fig. 5:**
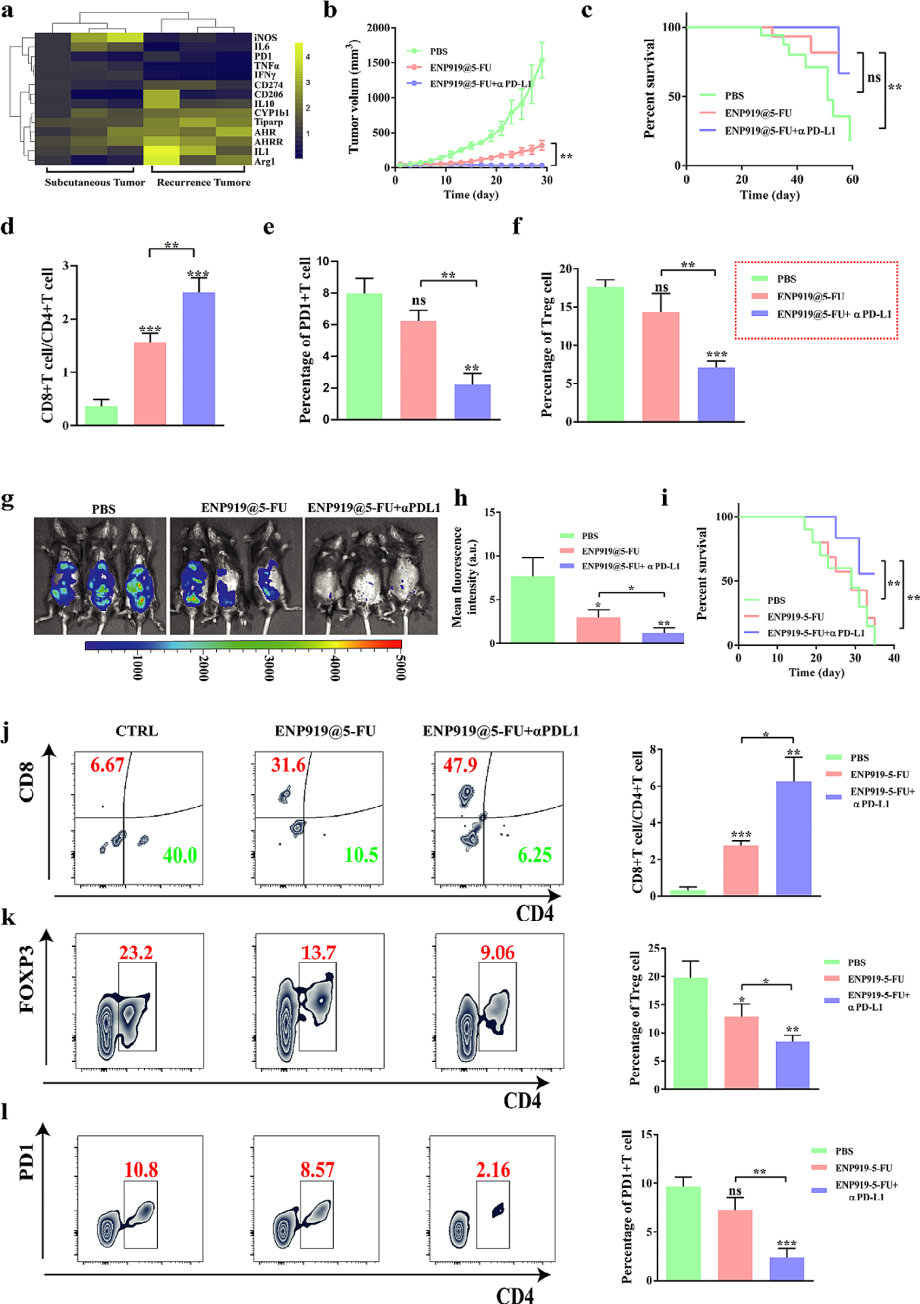
ENP919@5-FU combined with αPD-L1 significantly inhibited tumor recrudescence and metastasis in mice. (**a**) qPCR detected gene expression of subcutaneous tumors and recurrent tumors in mice of ENP919@5-FU group. (**b**-**c**) Subcutaneous tumor volume and survival analysis of Panc02-bearing C57 mice. (**d**-**f**) Effects of ENP919@5-FU combined αPD-L1 on immune cell infiltration in Panc02-bearing C57 mice. (**g**-**h**) Pancreatic cancer abdominal metastasis in C57 mice and fluorescence intensity analysis. (**i**) Effect of ENP919@5-FU + αPD-L1 on the survival of Panc02 mice with abdominal cavity metastasis. (**j**-**l**) Effects of ENP919@5-FU + αPD-L1 on immune cell infiltration in the ascites of C57 mice with abdominal metastases. Statistical analysis was performed by unpaired *t*-test and one-way ANOVA, *, *p* < 0.05; **, *p* < 0.01; ***, *p* < 0.001; ****, *p* < 0.0001

In previous experiments, although ENP919@5-FU showed excellent tumor suppression ability, tumor recrudescence was still observed. To explore strategies for preventing the occurrence of tumor recrudescence, we compared the gene expression of mice subcutaneous tumors and recurrent tumors in ENP919@5-FU group by qPCR detection. Unfortunately, the Kyn-AHR axis was reactivated in recurrent tumors, indicated with immunosuppressive microenvironment recurrence, i.e., the expression level of PD-1/PD-L1 was up-regulated [38] (Fig. 5a). Given the high expression of PD-1/PD-L1 in recurrent tumors, we supposed that the combination of ENP919@5-FU and αPD-L1 may have the effect of inhibiting tumor recrudescence.

Currently, immunotherapy, such as anti-PD-L1, has achieved remarkable success in a number of malignancies. However, due to the immune-suppressive tumor microenvironment, the therapeutic efficacy of anti-PD-(L)1 in pancreatic cancer is far from expectation [39]. Therefore, a pilot experiment was performed and suggested that anti-PD-L1 blockade alone has no significant effect on mice subcutaneous tumors (Fig. S21). In other words, in peritoneal metastasis models, free anti-PD-L1 blockers do not interfere with tumor treatment.

As shown in Fig. 5b and c, ENP919@5-FU combined with αPD-L1 could effectively inhibit the tumor growth and prolonged the survival period of mice, compared to ENP919@5-FU alone. Pleasantly, we found that united therapy could not only remarkably facilitate the differentiation of tumor-infiltrating lymphocytes into CD8 + T cells, but also reduced the proportions of negative indicators, illustrated as Treg cells and PD1 + T cells (Fig. 5d, e and f). Previous studies have confirmed that the concomitant appearance of low expression level of PD-L1 and inhibitory immune microenvironment will impair the effect of αPD-L1 in PDAC [40]. More encouragingly, our research suggested that ENP919@5-FU could also strengthen the efficacy of immune checkpoint inhibitors, reduce the expression of PD1 in tumor infiltrated lymphocytes (TILs), and improve the suppressive immune microenvironment of PDAC.

Abdominal implantation is an important metastasis pathway of PDAC [41, 42]. Undoubtedly, exploring strategies for metastatic tumors will greatly improve the current status of PDAC treatment. In addition, lone PD1/PD-L1 blocking therapy strategy is difficult to significantly improve the prognosis of PDAC, so it is necessary to seek a suitable model of combination treatment. Firstly, the peritoneal metastasis model of PDAC in C57 mice was successfully established, and the treatment process was implemented according to the schematic diagram (Fig. S22a). Intuitively found that the intraperitoneal in vivo fluorescence signal of Luc-Panc02 tumor-bearing mice was reduced by 7 times after the treatment of ENP919@5-FU + αPD-L1, compared to the control group, while the ENP919@5-FU alone was only one-half, indicating that ENP919@5-FU and αPD-L1 union could effectively subside the tumors in the abdominal cavity (Fig. 5g and h). Furthermore, the survival rate of mice was also greatly improved after combined therapy administration, compared to a single ENP919@5-FU treatment mode (Fig. 5i).

As mentioned above, the recurrence of suppressive immune microenvironment may be involved in tumor recurrence, which will also become a breakthrough point for improving the efficacy of metastatic tumors. Therefore, the combination therapy promoted the differentiation of CD8 + T cells in peritoneal metastasis mice and inhibited the differentiation of Treg cells and the expression of PD1 in T cells [43] (Fig. 5j, k and l). As shown in Fig. S22, there were more carcinogenesis and abnormal liver morphology in the control group. Luckily, after the union αPD-L1 therapy, the inhibition of metastatic tumors by ENP919@5-FU was significantly increased and the number of cancerous nodules in the liver decreased significantly. H&E results of neonatal nodules in the live were re-validated, and the ENP919@5-FU + αPD-L1 achieved an ideal therapeutic result. Additionally, the removal of important organs from the different treated-group of mice, H&E staining showed that ENP919@5-FU + αPD-L1 had a strong bio-safety (Fig. S23).

Overall, these experiments confirmed that ENP919@5-FU combined with αPD-L1 could inhibit the growth of PDAC metastases in mice through rebuilding the suppressive immune microenvironment of metastases to amplify chemoimmunotherapy.

## Conclusions

At present, due to the low solubility, lack of biological specificity, as well as easy to induce TIM formation, the application of 5-FU is quite limited in the PDAC. Therefore, a MMP-2/GHS dual-responsive liposome-based nanovesicle (ENP919@5-FU) was constructed, mainly consisting of MMP-2-responsive mPEG-GG-PPA, containing S-S linoleic acid-embellished NLG919 and 5-FU anti-cancer drug, for circulating chemoimmunotherapy amplification modes to effectively treat PDAC by reversing chemotherapy-mediated inhibition of the TIM. In the therapeutic process, the ENP919@5-FU nanovesicle was easily accumulated in tumor tissue by high expression MMP-2 enzymes, effectively enhancing tumor cellular uptake. Meanwhile, NLG919 and 5-FU were persistently released from nanovesicle *via* GSH reaction and effectively induced ICD effects by exposed 5-FU, i.e., activation the biological immune systems. However, the secretion of IFN-γ from effective T cell would re-establish Kyn-AHR axis based TIM, resulting in poor efficiency of tumor treatment. Interestingly, released IDO1 inhibitor NLG919 would distinctly reverse TIM by inhibiting the Kyn-AHR axis, further realizing intelligent nanovesicle-mediated chemoimmunotherapy cycle amplification in PDAC. Additionally, ENP919@5-FU nanovesicle combined with αPD-L1 to effectively and reasonably prevent tumor recurrence, which provides a simple but promising approach for advanced malignancies.

## Experimental procedures

### Material and regents

Fmoc-GPLGLAG-NH_2_ was synthesized by GL Biochem. Co., Ltd (Shanghai, China). 4-Dimethylaminopyridine (DMAP), 2-hydroxyethyl disulfide, 1-ethyl-3-(3-dimethylaminopropyl)carbodiimide hydrochloride (EDCI), 1-hydroxybenzotriazole (HOBT) and 4-Methylpiperidine were purchased from J&K Chemical (Shanghai, China). Methoxypolyethylene glycol amine (mPEG_5k_-NH_2_) was obtained from Sigma-Aldrich, USA. Pyropheophorbide A (PPa, 95%) was purchased from Dibai ChemTech Co., Ltd (Shanghai, China). 1,2-dipalmitoyl-sn-glycero-3-phosphorylcholine (DPPC), 1,2-dioctadecanoyl-sn-glycero-3-phophocholine (DSPC), and 1-cyclohexyl-2- (5 H-imidazo[5,1-a]isoindol-5-yl) ethanol (NLG919, 99%) were purchased from A.V.T Pharmaceutical Co., Ltd (Shanghai, China). Matrix metalloproteinase-2 (MMP-2) and 5-Fluorouracil (5-FU) was obtained from Meilunbio (Shanghai, China). Linoleic acid and reduced glutathione (GSH, 98%) were bought from Shanghai Chemical Factory (Shanghai, China). N, N-dimethylformamide (DMF), dimethyl sulfoxide (DMSO) and ethanol were obtained from Beijing Chemical Reagents Company (Beijing, China). 2′,7′-dichlorodihydrofluorescein diacetate (DCFH-DA) was obtained from Sigma-Aldrich Co. (St. Louis, MO, USA). Cell Counting Kit-8 (CCK-8) assay kit was purchased from Beyotime Biotechnology. Phosphate-buffered saline (PBS), penicillin-streptomycin (100 ×) and Dulbecco’s modified Eagle’s medium (DMEM) were obtained from Life Technologies Corporation (Los Angeles, CA, USA). Standard fetal bovine serum (FBS) was purchased from ExCell Bio. The ultrapure water (18.2 MU, Milli-Q, Millipore) was used throughout the experiments. All reagents and chemicals were used without further purification.

### Synthesis of NLG919-SS-linoleic acid (N-SS-LA)

The reacted ultra-dry DCM (15 mL) solution containing 200 mg of linoleic acid (LA, 0.71 mM), 410 mg of 1-ethyl-3-(3-dimethylaminopropyl) carbodiimide (EDCI, 2.14 mM), 260 mg of 4-dimethylaminopyridine (DMAP, 2.14 mM) was stirred under N_2_ condition. The addition of 760 µL of DIEA into mixed solution then reacted for 1 h. After that, the reacted solution added 330 mg of 2-hydroxyethyl disulfide (2.14 mM) to continue the reaction for 24 h at room temperature. Finally, the reaction solution was washed thrice with ammonium chloride aqueous solution, and collected organic phase and dried with sodium sulfate. The purified product of LA-SS-OH was obtained by column chromatography method (yield, 170 mg, 57%).

The NLG919 (50 mg, 0.18 mM), DMAP (110 mg, 0.9 mM) and triphosgene (21 mg, 0.07 mM) were dissolved into 5 mL of ultra-dry DCM in an ice bath and reacted under N_2_ atmosphere for 1 h. Subsequently, the reacted solution was added LA-SS-OH (74 mg) and reacted for 24 h. Finally, the reaction solution was washed thrice with an aqueous ammonium chloride solution. The organic phase was collected, dried with sodium sulfate, filtered to obtain the filtrate, rotary evaporated to remove the solvent and column separated to obtain the product (NLG919-SS-linoleic acid (N-SS-LA), 80 mg) with a yield of 62%.

### Synthesis of mPEG5k-GPLGLAG-PPa (P-GG-Pa) and mPEG5k-PPa (P-Pa)

EDCI (71.4 mg, 0.372 mM), and HOBT (50.2 mg, 0.272 mM) and fmoc-GPLGLAG-NH_2_ (200 mg, 0.23 mM), were dissolved in 5 mL DMF and stirred for 1 h at room temperature to activate the carboxyl group. Immediately after, 2 mL of DMF liquor containing mPEG-NH_2_ (1.0 g, Mn = 5000, 0.2 mM) slowly added dropwise to the above reacted system, and stirred for 24 h at room temperature. Then, 1.4 mL 4-methylpiperidine was added again and reacted for 12 h. Finally, the light-yellow powder (named mPEG_5k_-GPLGLAG-NH_2_) was obtained via precipitating with ice ether and lyophilization.

The 5 mL DMF solution containing mPEG_5k_-GPLGLAG-NH_2_ (1.0 g) or mPEG_5k_-NH_2_ (877 mg) slowly added to 5 mL DMF solution containing pyropheophorbide A (PPa, 200 mg, 0.365 mM), EDCI (71.4 mg, 0.372 mM) and HOBT (50.2 mg, 0.272 mM), and stirringly reacted for 24 h at room temperature. The crude product was collected by precipitating with ice ether, and centrifuging at 5000 rpm/10 min. Later, the crude product was purified by column chromatography method (acetonitrile: deionized water (containing 0.1‰ trifluoroacetic acid) = 7 : 3), named as mPEG-GPLGLAG-PPa (P-GG-Pa).

At the same time, according to the above preparation method of P-GG-Pa, the PEG_5k_-PPa (P-Pa) was obtained and refrigerated for use.

### Nanovesicle preparation

The plural nanovesicle were constructed according to previous the literature [34]. Simply, different samples with a concentration of 1 mg/mL chloroform solution were pre-prepared, involving DPPC, DSPC, N-SS-LA and P-GG-PA, meanwhile, 5-FU solution was prepared as a 10 mg/mL aqueous solution. Then, the volume ratios of DPPC : DSPC : N-SS-LA : P-GG-PA : 5-FU = 3:1:1:3:0.8 (v/v) were mixed by ultrasonic for 20 min to generate a water-in-oil mixed system. After that, the homogeneous film was formed via the pressure rotary evaporator at 50 °C, and liposome colostrum was obtained following hydration at 55 °C for 1 h. Eventually, the nanovesicle (ENP919@5-FU ) with suitable particle size were collected through continuously extruding 21 times with 200 nm and 100 nm filters. The NP919@5-FU and NP919 nanovesicle also were obtained according to similar programme.

### Characterization

The morphology of nanovesicle was characterized by JEM-2100 F transmission electron microscope (TEM, JEOL, Tokyo, Japan). The mean diameter and zeta potential of the as-prepared nanovesicle was measured by the dynamic laser light scattering (DLS, ZEN 3600, Malvern Instruments). The release profile of 5-FU and NLG919 were tested by high performance liquid chromatography (Waters, USA). 1 H NMR spectra were characterized with a Nuclear Magnetic Resonance instrument (JEOL ECS 400 M).

### 5-FU release

An 18 C reverse chromatographic column was used to detect 5-FU. The mobile phase comprised water-methanol (95:5); the flow rate was 1.0 mL/min, whereas the detection wavelength was 265 nm. The injection volume was 20 µL. The peak time was approximately 4.0 min.

### Cells and animals

Panc02 cell line and Luc-Panc02 cell line were purchased from Shanghai Cell Bank of the Chinese Academy of Sciences. MMP-2-KD Panc02 cells were conducted by ABIOCENTER Company. The cells were cultured in a DMEM medium containing 10% FBS and 1% penicillin-streptomycin in a 5% CO_2_ incubator at 37 °C. All C57 mice (aged 6–8 weeks) were purchased from Shanghai B&K Company and raised under SPF standards. All animal experiments were approved by the institutional ethics committee of Shanghai Jiaotong University and all procedures were performed according to the rules of the Institutional Animal Care and Use Committee of Shanghai Jiaotong University (ICCUC NO. 2020-09-XLM-03).

### Panc02 subcutaneous tumor model

Sixty female C57 mice (aged 6–8 weeks) were purchased from Shanghai B&K Company and raised under SPF standards. Each mouse received a subcutaneous injection of Panc02 cells suspension (1 × 10^6^ cells /mouse) on the back of the hind limbs, and the tumor volume grew to around 50 mm^3^ before used. Subsequently, these mice were divided randomly into 5 groups, i.e., PBS, ENP919, 5-FU, NP919@5-FU, and ENP919@5-FU. On the 1st, 4th, and 7th days, mice in each group were injected with an equal volume of PBS or different samples (containing 5-FU: 50 mg/kg or NLG919: 16 mg/kg) *via* the tail vein, respectively. Then, five mice from each group were sacrificed randomly at 15th day, and tumors and tumor-draining lymph nodes were collected for further investigation. The remaining mice continued to observe and document tumor volume and the animal survival. If the tumor volume exceeded 2000 mm^3^ or the tumor was ulcerated during the observation period, the animal was deemed dead. All surviving mice were sacrificed after 60 days.

The Panc02 subcutaneous tumor model was used to validate the tumor-suppressive effect of ENP919@5-FU combined with αPD-L1, and mouse model was developed. The feeding method remained the same. Thirty-six tumor-bearing mice were divided into 3 groups: PBS, ENP919@5-FU and ENP919@5-FU combined with αPD-L1 (containing 50 mg/kg 5-FU, αPD-L1 100 µg/mouse). Tumor immunoassay and survival analysis were performed after three administrations. The administration and monitoring procedures remained unchanged.

### Panc02 abdominal cavity metastasis model

Thirty female C57 mice (6–8 weeks) were raised in an SPF environment before being intraperitoneally injected with 3 × 10^6^ Luc-Panc02 cells. The mice were randomly divided into three groups on the second day after tumor injection. Mice in each group were injected intraperitoneally with PBS, ENP919@5-FU, and ENP919@5-FU + αPD-L1 (the same dose as before) once every 2 days. On the 15th day, 3 mice from each group were randomly selected for live imaging and dissection, and ascites from each mice were collected for immunological analysis. The remaining mice were raised and their survival conditions were documented.

### Live imaging

Abdominal metastasis model mice constructed by Luc-Panc02 cells were depilated on the chest and abdomen. Mice were anesthetized with isoflurane, and injected d-fluorescein sodium salt (MB18371-1, Meilun) 5 mg/mouse into the abdominal cavity and fluorescence imaging was performed. Fluorescence imaging was performed 15 min later, and the fluorescence value was statistically analyzed.

Ten 4-week-old female nude mice were injected subcutaneously with 1 × 10^6^ Panc02 cells on the dorsal side of the hind limbs. When the tumor volume reached 100 mm^3^, the mice were randomly divided into NP919@5-FU group and ENP919@5-FU group. NP919@5-FU and ENP919@5-FU (including PPa 5 mg/kg) were injected *via* the tail vein. Small animal imaging was used to examine the accumulation of nanovesicle in the tumor at 2, 4, 12, 24, and 48 h after injection. The mice were sacrificed after 48 h, and the liver, kidney, and tumor were obtained for imaging.

### qPCR technology

The RNA extraction kit (19211ES60, Yeasen) was used to extract RNA from cells and animal tissues. RNA was reverse transcribed to cDNA (11121ES60, Yeasen) for qPDACR experiments (11201ES03, Yeasen). The following is the primer sequence:

### ELISA measurement

ELISA kits were used to examine the content of Kyn (PM104687) and Trp (PM103881) in cell supernatants and tumor tissues using the manufacture’s protocol.

**Table:** 

Gene	Forward	Revise	Gene	Forward	Revise
IFN-γ	ATGGCTAGGCCCTTTGCTTTC	ACCAGTAGCTTATCACCACCA	IL1	AGCAGTCCCAACTAAGCAGTA	CAGCCAGTAGAGGATGCTGA
CYP1b1	CCACCAGCCTTAGTGCAGAC	GGCCAGGACGGAGAAGAGT	IL10	TTGTCGCGTTTGCTCCCATT	GAAGGGCTTGGCAGTTCTG
Tiparp	CACCCTCTAGCAATGTCAACTC	CAGACTCGGGATACTCTCTCC	Arg1	TGTCCCTAATGACAGCTCCTT	GCATCCACCCAAATGACACAT
AHR	GACCCTCCTCAGGTGGTGTT	AATGAAGCAGCGTGTCAAGAA	CD206	TGGCCTCTGAGTTTAGGGTCT	CCCTTGGTGTCGAACCAGC
AHRR	GACCCTCCTCAGGTGGTGTT	AATGAAGCAGCGTGTCAAGAA	iNOS	CTCTTCGACGACCCAGAAAAC	CAAGGCCATGAAGTGAGGCTT
TNF-α	GCGGCCACAGAAAACACTC	CTCCCAATGGTCAAGGCATC	PD1	AACCGCACAGTCACGCTAC	TGATCGTCAATTCCCGGAACA
IL-6	TGGGGCTCTTCAAAAGCTCC	AGGAACTATCACCGGATCTTCAA	CD274	GCTCCAAAGGACTTGTACGTG	TGATCTGAAGGGCAGCATTTC

### Bone marrow mononuclear cells (BMNC) extraction and induction

BMNC were extracted from C57 mice aged 6–8 weeks using the method described in the literature [44]. The obtained BMNCs were cultured in a DMEM medium containing 10% FBS, C-GSF (20 µg/mL), and IL4 (20 µg/mL) for 7 days to obtain bone marrow dendritic cells (BMDC). Panc02 cells were treated by control, ENP919, 5-FU, ENP919@5-FU and NP919@5-FU (contained 5-FU 20 mM) for 24 h, then BMDC and Panc02 cells were co-incubated for another 24 h, and the expression of CD11c, CD80, and CD86 in BMDC were detected by a flow cytometer.

### Flow cytometer (FCM)

FCM was used to detect the expression of biomarkers on CD4/CD8 T cells, Treg cells in mice tumor; DC and central memory T cells in mouse tumor-draining lymph nodes. BD Fortessa flow cytometer (BD Biosciences) was used for flow cytometric measurement and Flowjo was used to data analysis. The antibodies that were used are listed in the table below.

**Table:** 

Cells	Antibodies
CD4 T cells	Anti-CD45, Anti-CD3, Anti-CD4
CD8 T cells	Anti-CD45, Anti-CD3, Anti-CD8
PD1 + T cell	Anti-CD45, Anti-CD3, Anti-PD1
IFN-γ CD8 T cells	Anti-CD45, Anti-CD8, Anti-IFN-γ
Dendritic cells	Anti-CD11c, Anti-CD80, Anti-CD86
Treg cells	Anti-CD45, Anti-CD3, Anti-CD4, Anti-FOXp3
Central memory T cells	Anti-CD45RA, Anti-CD4, Anti-CD62L

Tumor infiltrated lymphocytes (TILs) were extracted from tumor tissue. The final concentrations of hyaluronidase, collagenase VI, and DNase in DMEM medium were 1.0 mg/mL, 1.0 mg/mL and 0.5 mg/mL, respectively. Tumor tissue were cut into pieces and incubated in 5 mL of culture medium at 37 °C and grinded once every 30 min for a total of three times. The resultant suspension was subjected to density gradient centrifugation to obtain TILs.

### Immunofluorescence (IF), H&E staining, and immunocytochemistry (IHC)

The Panc02 cell slides were fixed with 4% paraformaldehyde, and IF was used to detect AHR expression in the cells. For subsequent experiments, tumor tissues were fixed in 4% paraformaldehyde and cut into paraffin sections. H&E staining was used to characterize tumor tissue. IHC was used to assess AHR expression in tumors. IF was used to evaluate the liposome uptake by tumor cells.

### Statistical analysis

All experiments were repeated for 3 times. Data were presented as mean ± standard deviation. Student’s *t*-test or one-way ANOVA was used to calculate statistical differences in Graphpad Prism 6.0 and *p* < 0.05 was considered statistically significant: *p < 0.05,**p < 0.01, and ***p < 0.001.

### Electronic supplementary material

Below is the link to the electronic supplementary material.

Supplementary Material 1

## Data Availability

No datasets were generated or analysed during the current study.
