# Supplementary material for: Intelligent nanovesicle for remodeling tumor microenvironment and circulating tumor chemoimmunotherapy amplification
Source: J Nanobiotechnology. 2024 May 16;22:257. doi: 10.1186/s12951-024-02467-8 (PMC11097415; doi:10.1186/s12951-024-02467-8)
Supplement: Supplementary file 1 — Supplementary Material 1 [file 12951_2024_2467_MOESM1_ESM.docx]

**Intelligent** **nanovesicle for remodeling tumor microenvironment and** **circulating tumor** **chemoimmunotherapy** **amplification**

*Manxiu Huai^1^, Yingjie Wang^2^, Junhao Li^3^, Jiaxing Pan^1^, Fang Sun^1^, Feiyu Zhang^1^, Yi Zhang^1^*，Leiming Xu^1^**


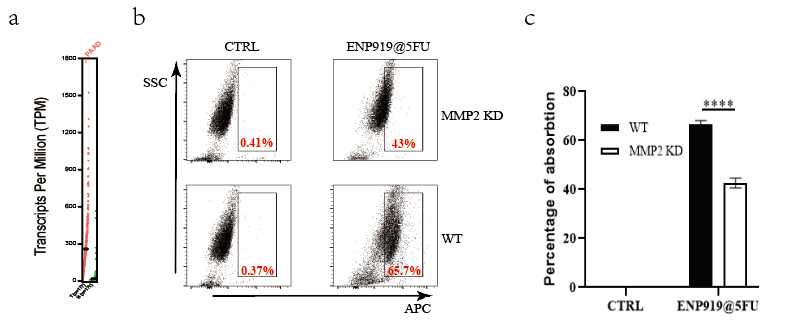


**Fig. S1**. The expression level of MMP-2 is correlated with the absorption rate of ENP919@5-FU in Panc02 cells. (a) Expression level of MMP-2 in pancreatic cancer tissues and adjacent tissues. (b-c) Compared with WT Panc02 cells, the uptake of ENP919@5-FU decreased in MMP-2 KD cells.


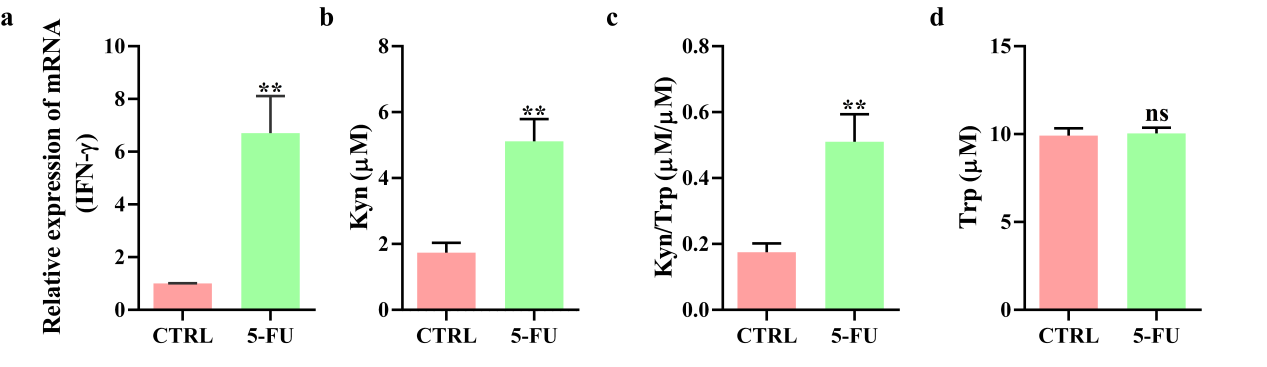


**Fig. S2.** 5-FU intervention influenced Trp metabolism in Panc02-bearing C57 mice. A) qPCR detection of IFN-γ expression in Panc02 tumor-bearing C57 mice. B) ELISA detection of Kyn levels in Panc02 tumor-bearing C57 mice. C) ELISA detection of Trp levels in Panc02 tumor-bearing C57 mice. D) The Kyn/Trp ratio in Panc02 tumor-bearing C57 mice. Statistical analysis was performed by unpaired *t*-test, *, *p* < 0.05; **, *p* < 0.01; ***, *p* < 0.001; ****, *p* < 0.0001.


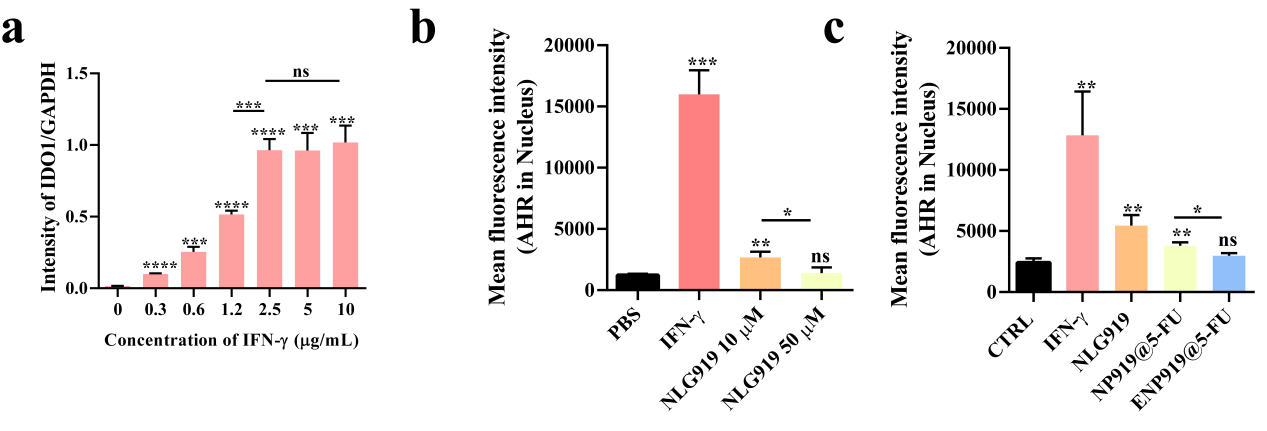


**Fig. S3.** Quantitative analysis of Panc02 cells. (a) Quantitative analysis of western blot bands of IDO1/GAPDH in Panc02 cells. (b, c) Quantitative analysis of relative fluorescence intensity of AHR in Panc02 cells by different sample treating.

**Fig. S4.** Synthetic route of LA-SS-OH and N-SS-LA.

**Fig. S5.** Synthetic route of mPEG_5k_-PPa (P-Pa).

**Fig. S6.** Synthetic route of mPEG_5k_-GALGLPG-PPa (P-GG-Pa).

**
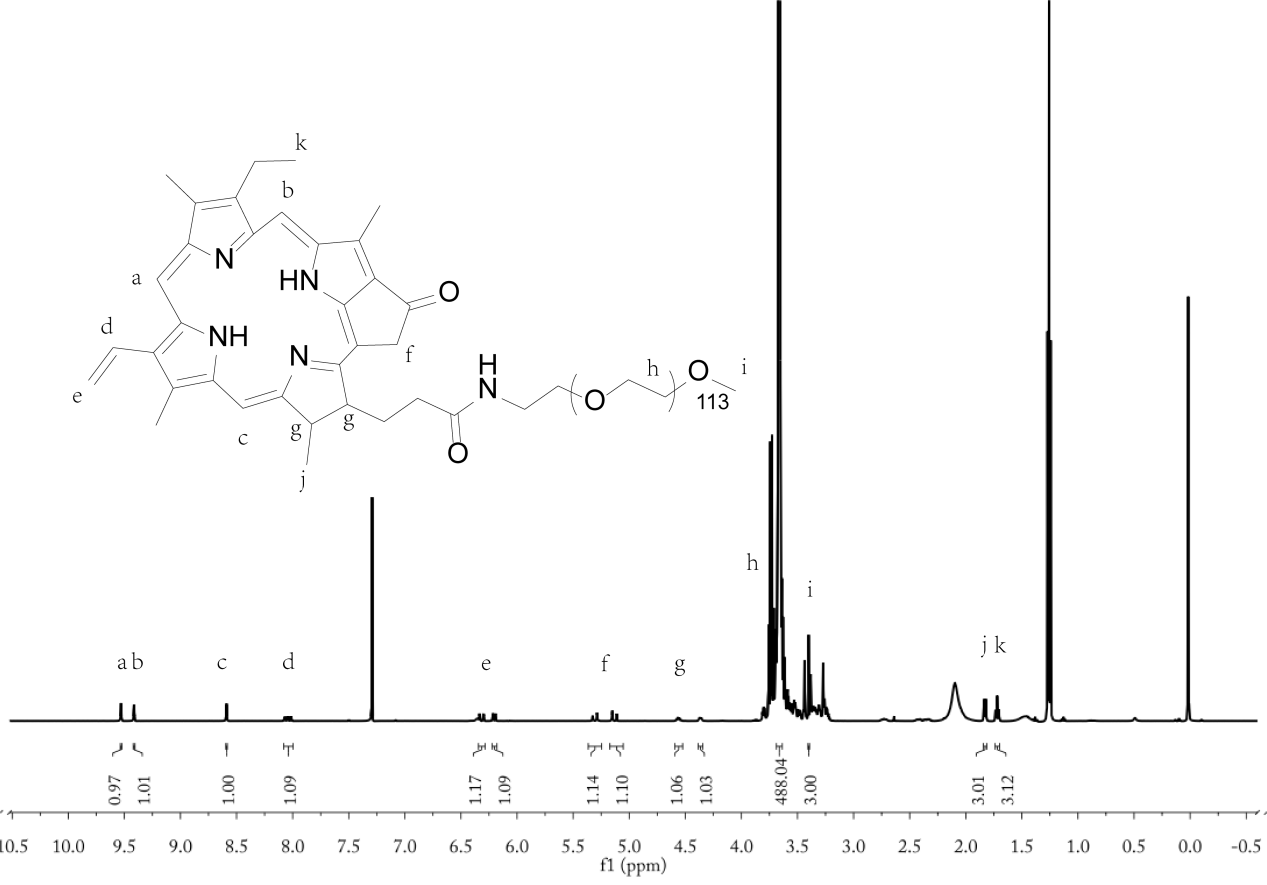
**

**Fig. S7.** ^1^H NMR spectra of P-Pa.

**
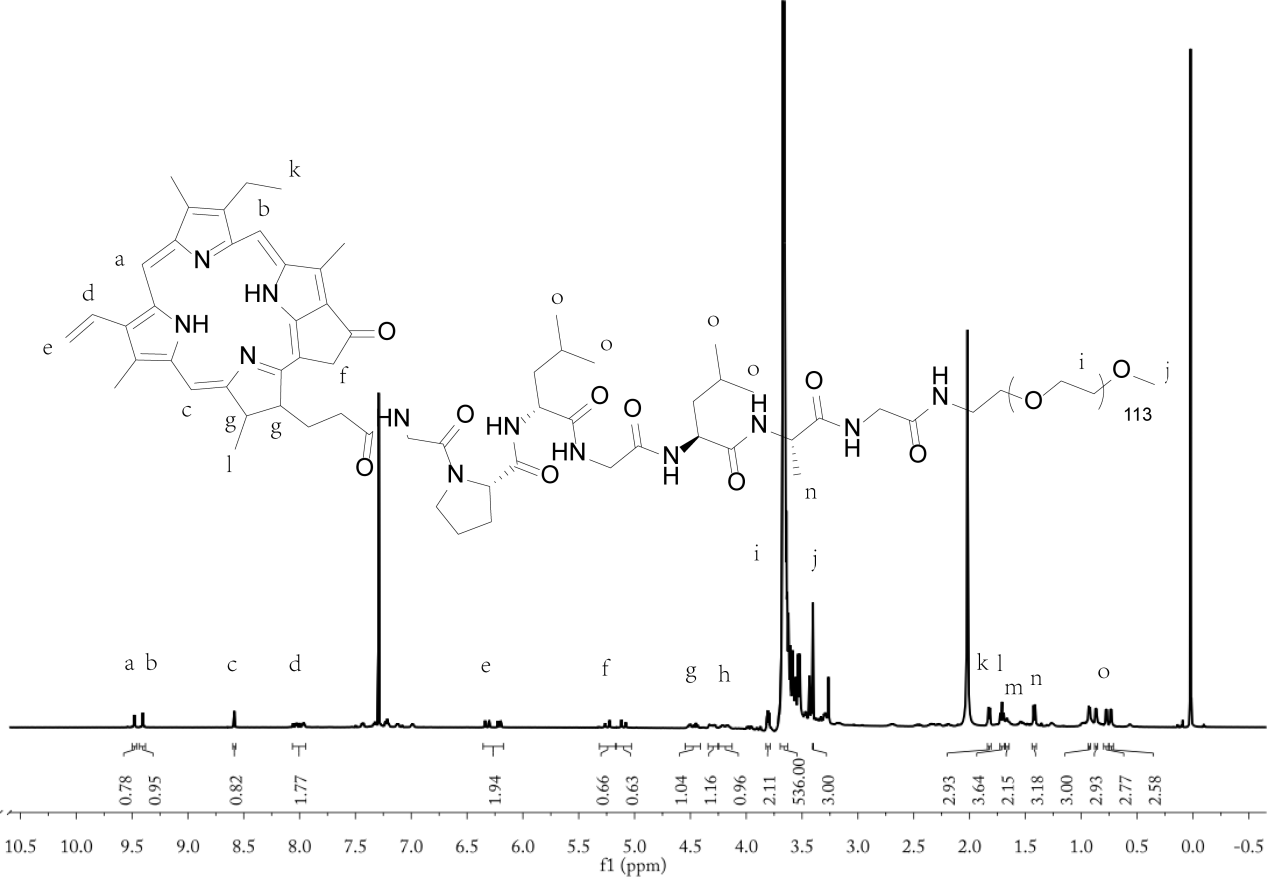
**

**Fig. S8.** ^1^H NMR spectra of P-GG-Pa.

**
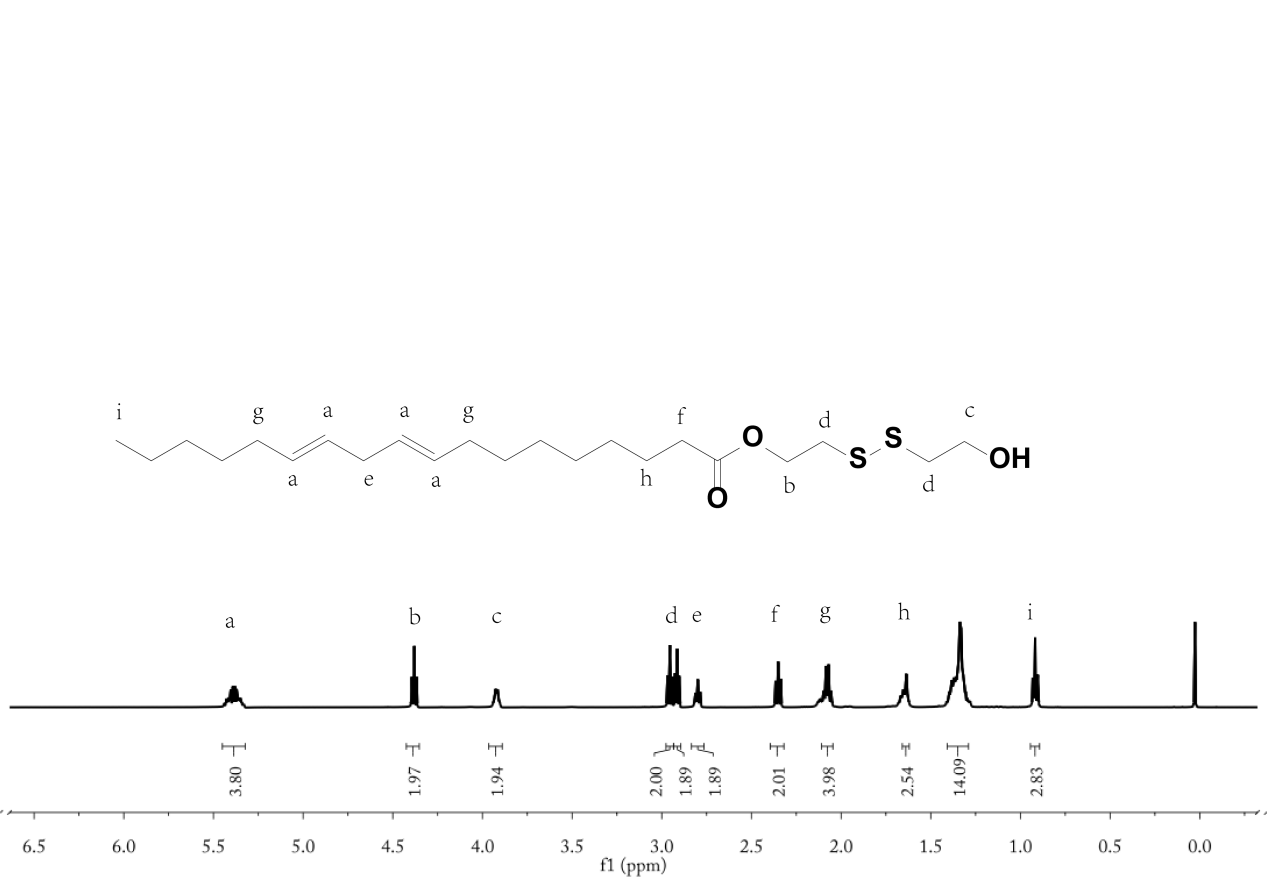
**

**Fig. S9.** ^1^H NMR spectra of LA-SS-OH.

**
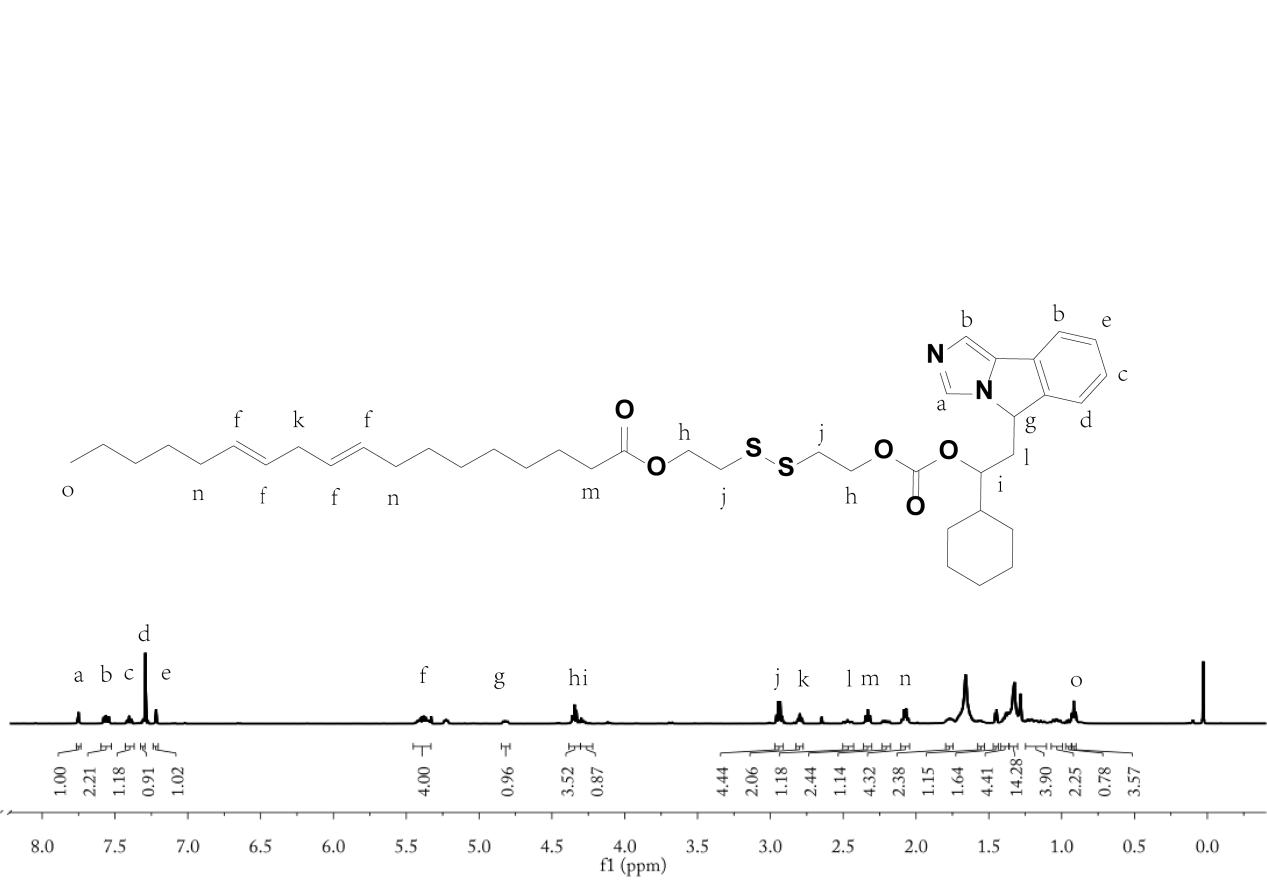
**

**Fig. S10.** ^1^H NMR spectra of LA-SS-N.





**Fig. S11.** The particle size and TEM image of NP919@5-FU.

**
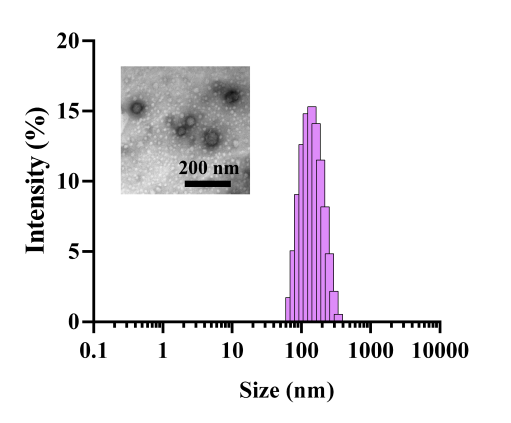
**

**Fig. S12.** The particle size and TEM image of ENP919.

**
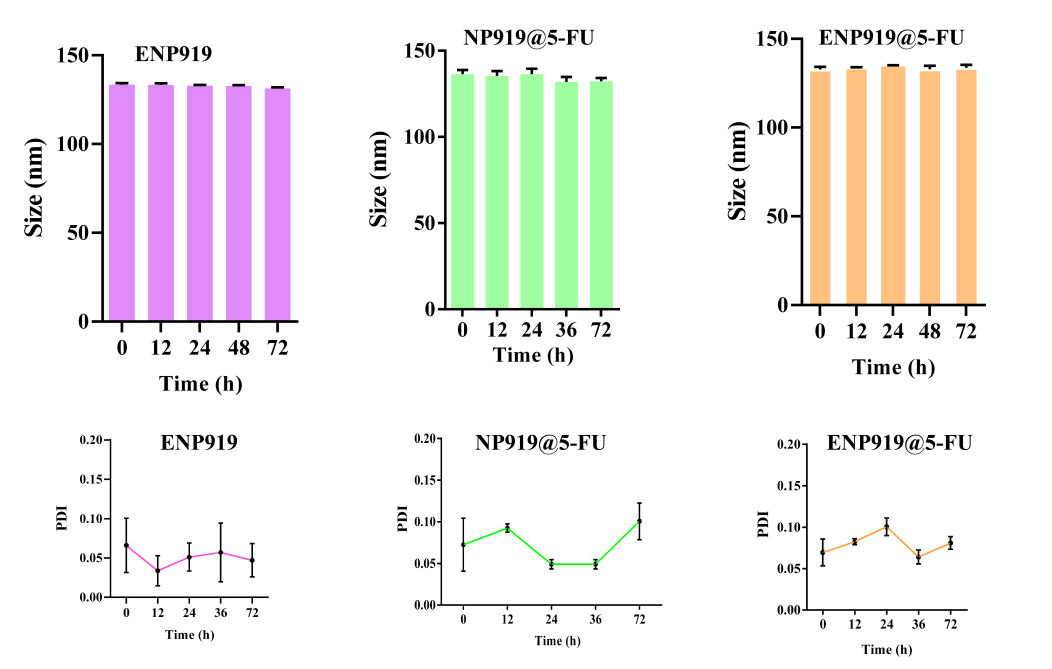
**

**Fig. S13.** Stability testing of ENP919, NP919@5-FU and ENP919@5-FU.

**
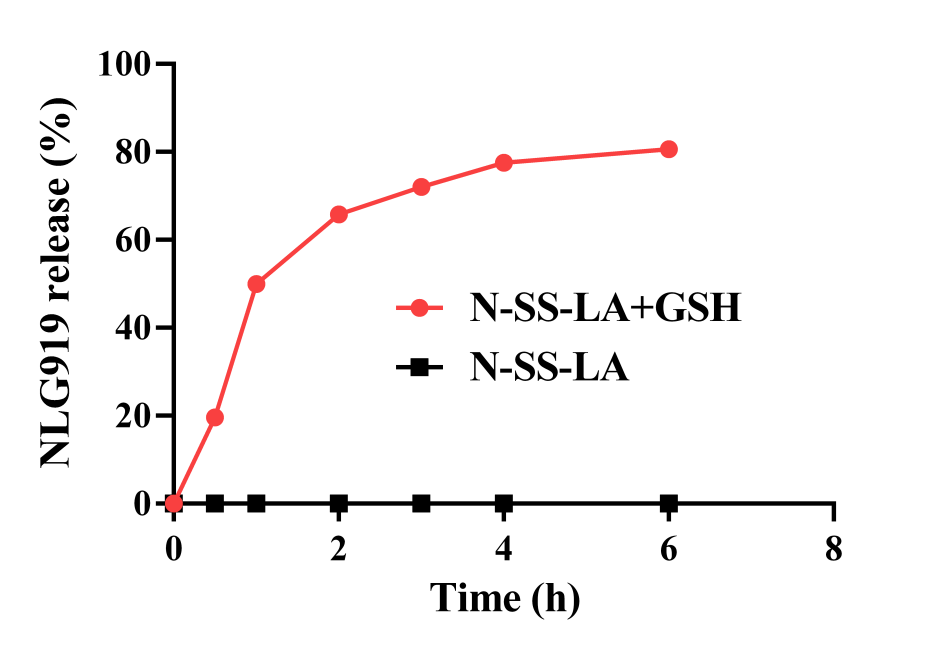
**

**Fig. S14.** The NLG919 release profile of N-SS-LA under10 mM GSH treatment by HPLC testing.


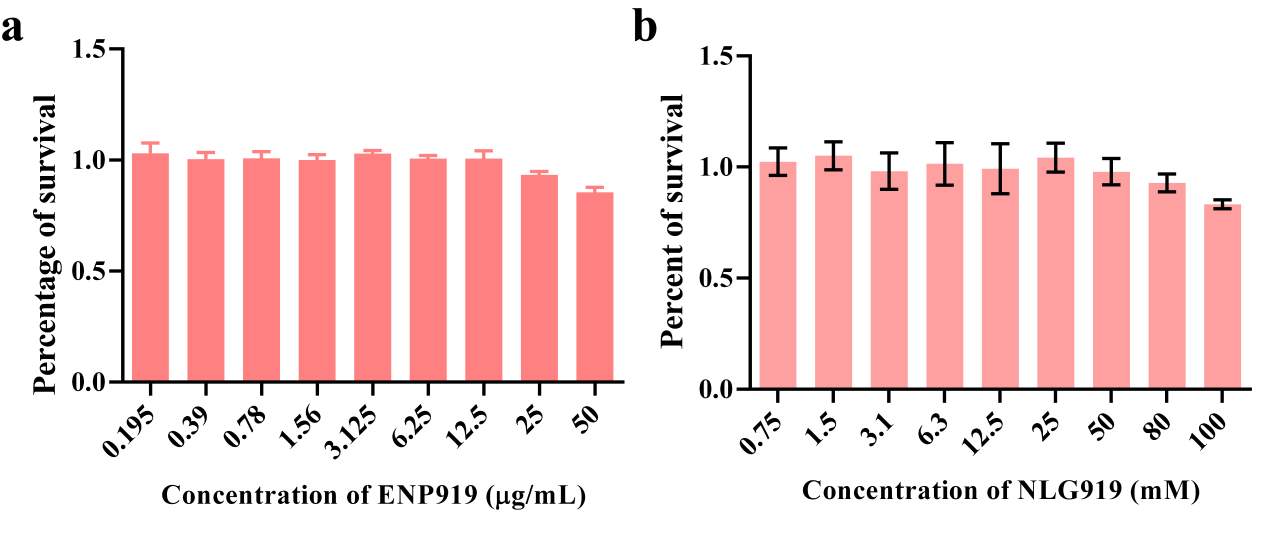


**Fig. S15.** The cell viability of ENP919 nanovesicle and free NLG919 by CCK-8 assay.


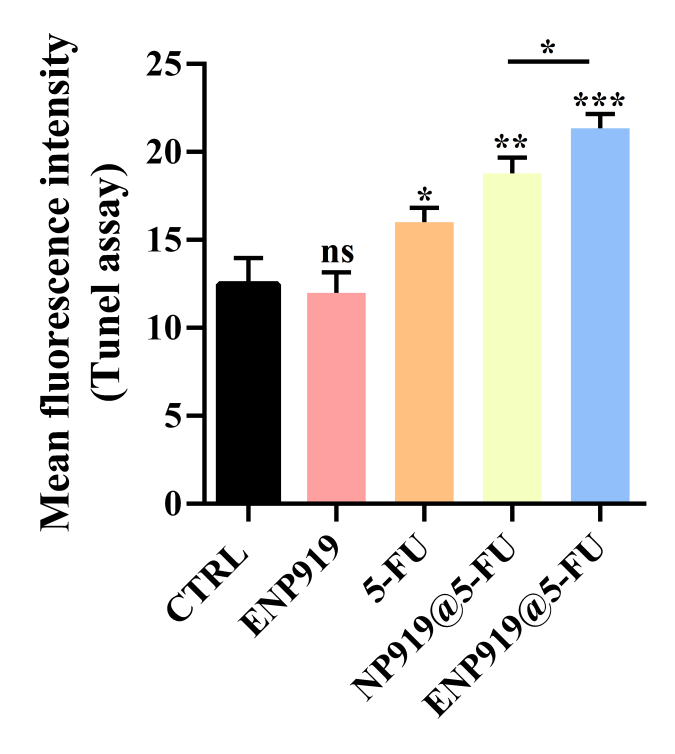


**Fig. S16.** Quantitative signal intensities of TUNEL-positive cells in Panc02 tumor slices.


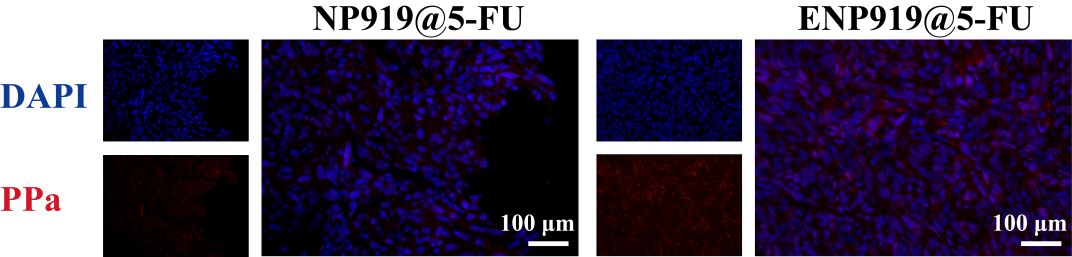


**Fig. S17.** Fluorescence detection of the enrichment of containing PPa probe NP919@5-FU and ENP919@5-FU in tumor tissues.
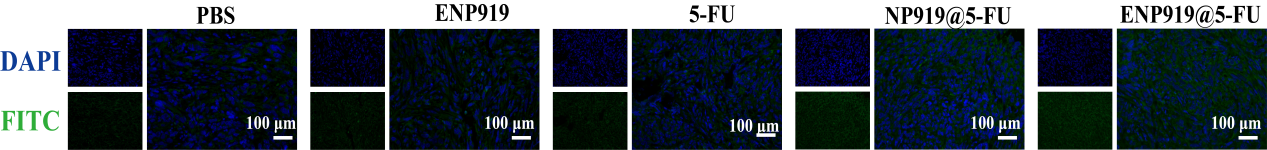


**Fig. S18.** Immunofluorescence detection of CRT effect in different samples-treated tumor tissues.


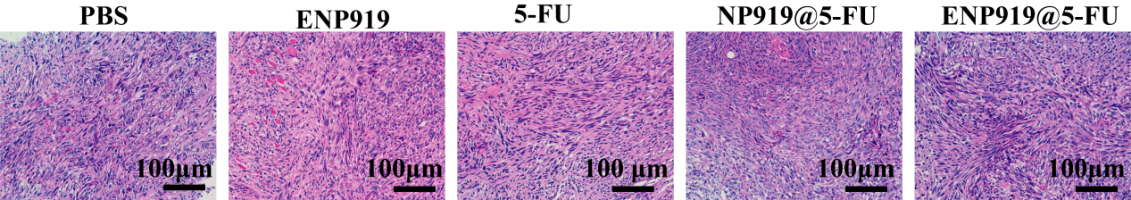


**Fig. S19.** H&E staining of Panc02-bearing C57 mice tumors by various samples treating.


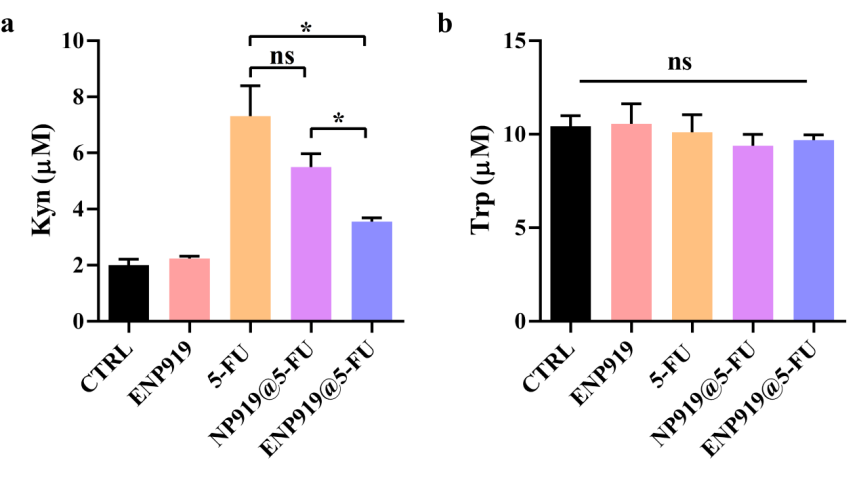


**Fig. S20.** The effect of nanovesicles on the expression of Kyn and Trp in Panc02-bearing C57 mice. (a) ELISA detection of the expression level of Kyn in Panc02-bearing C57 mice. (b) ELISA detection of the expression level of Trp in Panc02-bearing C57 mice. Statistical analysis was performed by unpaired *t*-test, *, *p* < 0.05; **, *p* < 0.01; ***, *p* < 0.001; ****, *p* < 0.0001.

*
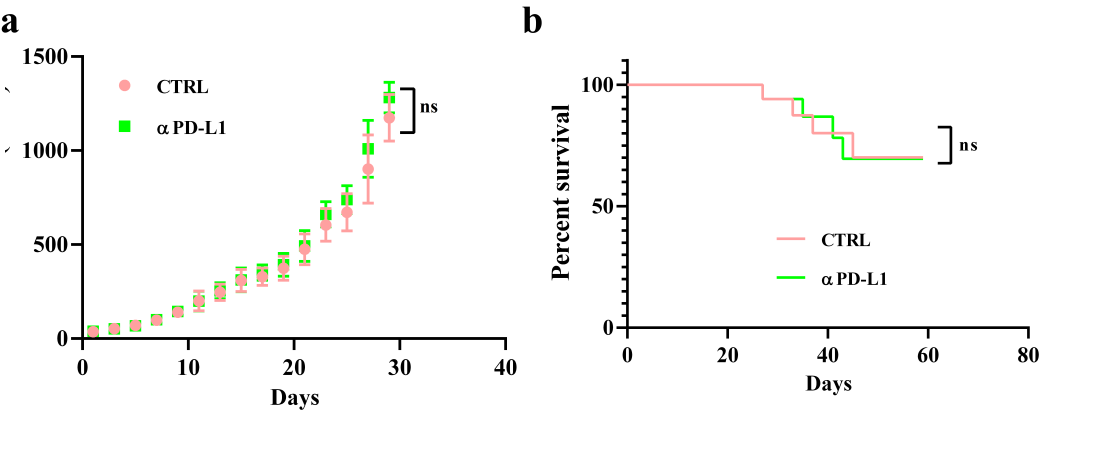
*

**Fig. 21.** The effects of anti-PD-L1 blockade alone in PDAC mice model. (a-b) The tumor volume and survival analysis of anti-PD-L1 blockade treatment.


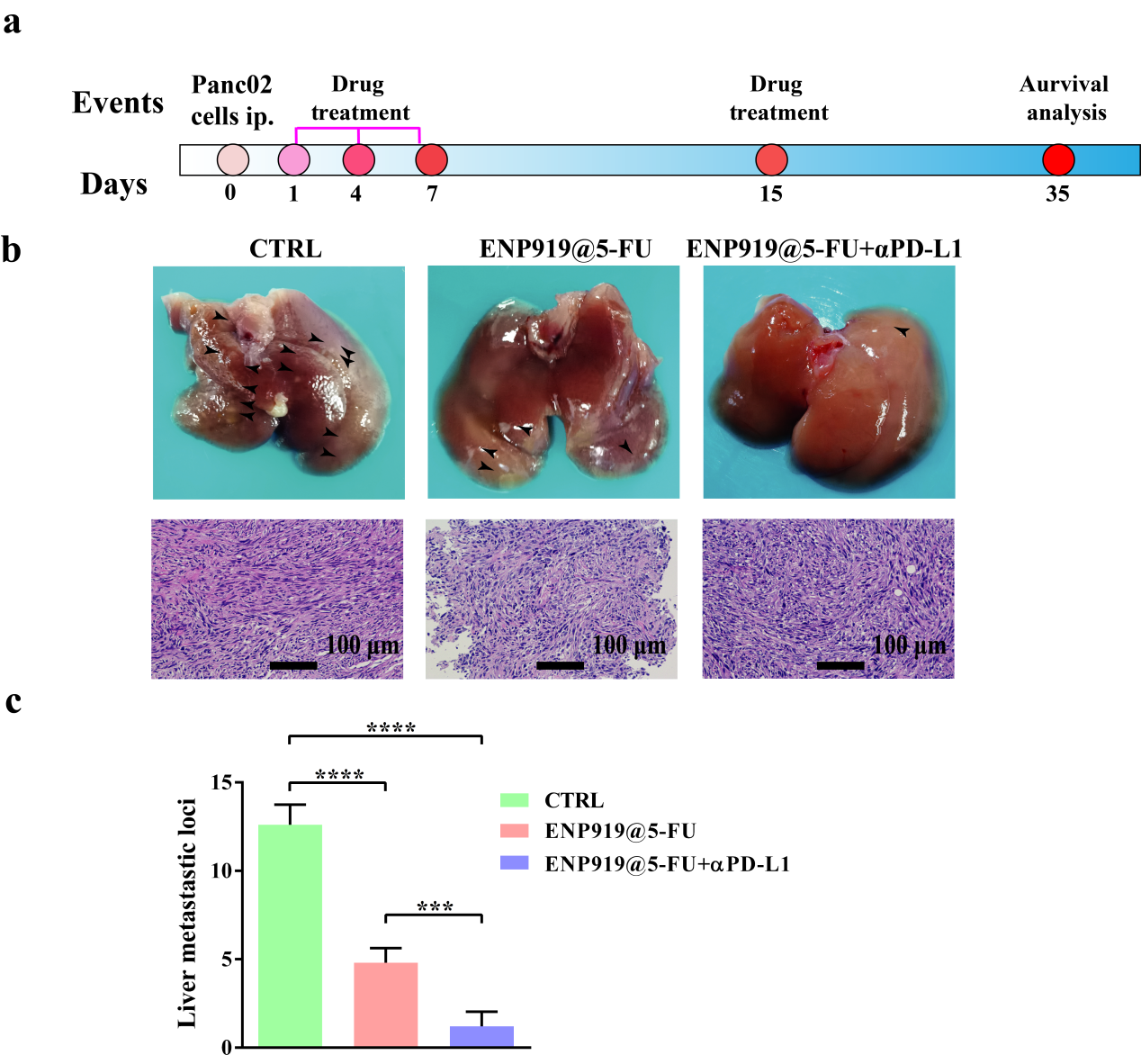


**Fig. S22.** (a) Thirty female C57 mice aged 6-8 weeks were reared in an SPF environment, and each mouse was intraperitoneally injected with 3×10^6^ Luc-Panc02 cells. (b) The mice were randomly divided into 3 groups: PBS, ENP919@5-FU, and ENP919@5-FU + αPD-L1 (containing 50 mg/kg 5FU, αPD-L1 100 μg/mouse), injected intraperitoneally on the 1^st^, 3^th^, and 5^th^ days after modeling. Three mice from each group were randomly selected on the 15^th^ day for live imaging and dissection. After that, abdominal metastases were collected for immunological analysis. The remaining mice were raised and their survival conditions were documented. H&E staining of metastatic tumors. Gross liver metastases in Panc02-bearing C57 mice were shown. (c) Analysis of the number of liver metastases in mice. Statistical analysis was performed by unpaired *t*-test, *, *p* < 0.05; **, *p* < 0.01; ***, *p* < 0.001; ****, *p* < 0.0001.


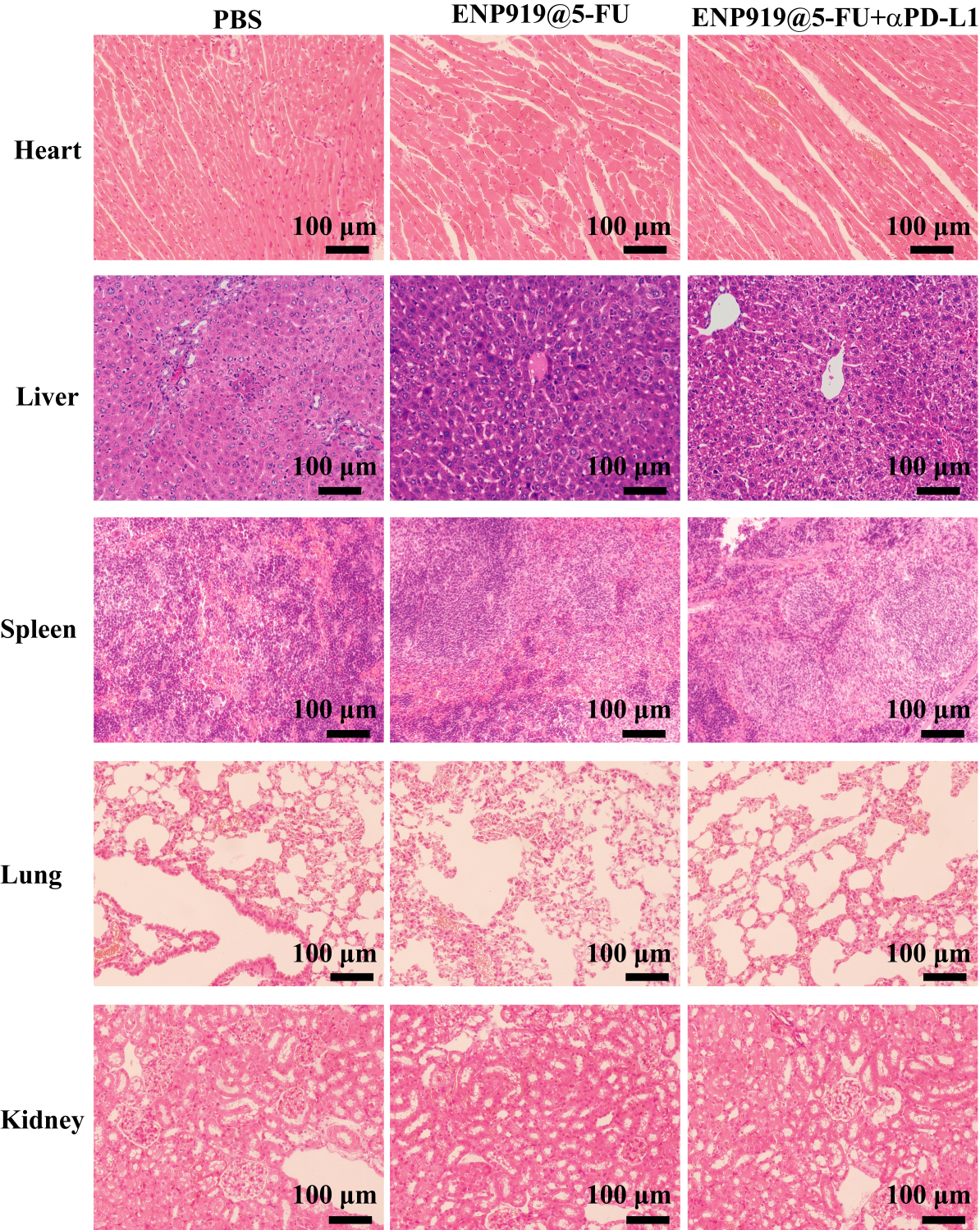


**Fig. S23.** H&E staining images of major organs, scale bar: 100 μm
